# Cholesterol trafficking to the ER leads to the activation of CaMKII/JNK/NLRP3 and promotes atherosclerosis

**DOI:** 10.1016/j.jlr.2024.100534

**Published:** 2024-03-22

**Authors:** Mustafa Yalcinkaya, Wenli Liu, Tong Xiao, Sandra Abramowicz, Ranran Wang, Nan Wang, Marit Westerterp, Alan R. Tall

**Affiliations:** 1 Division of Molecular Medicine, Department of Medicine, Columbia University Irving Medical Center, New York, NY, USA; 2 Department of Pediatrics, University Medical Center Groningen, University of Groningen, Groningen, The Netherlands

**Keywords:** cholesterol, NLRP3 inflammasome, ER, CaMKII, JNK

## Abstract

The deposition of cholesterol-rich lipoproteins in the arterial wall triggers macrophage inflammatory responses, which promote atherosclerosis. The NLRP3 inflammasome aggravates atherosclerosis; however, cellular mechanisms connecting macrophage cholesterol accumulation to inflammasome activation are poorly understood. We investigated the mechanisms of NLRP3 inflammasome activation in cholesterol-loaded macrophages and in atherosclerosis-prone *Ldlr*^*−/−*^ mice with defects in macrophage cholesterol efflux. We found that accumulation of cholesterol in macrophages treated with modified LDL or cholesterol crystals, or in macrophages defective in the cholesterol efflux promoting transporters ABCA1 and ABCG1, leads to activation of NLRP3 inflammasomes as a result of increased cholesterol trafficking from the plasma membrane to the ER, via Aster-B. In turn, the accumulation of cholesterol in the ER activates the inositol triphosphate-3 receptor, CaMKII/JNK, and induces NLRP3 deubiquitylation by BRCC3. An NLRP3 deubiquitylation inhibitor or deficiency of *Abro1*, an essential scaffolding protein in the BRCC3-containing cytosolic complex, suppressed inflammasome activation, neutrophil extracellular trap formation (NETosis), and atherosclerosis in vivo. These results identify a link between the trafficking of cholesterol to the ER, NLRP3 deubiquitylation, inflammasome activation, and atherosclerosis.

## Graphical abstract

**Figure:**
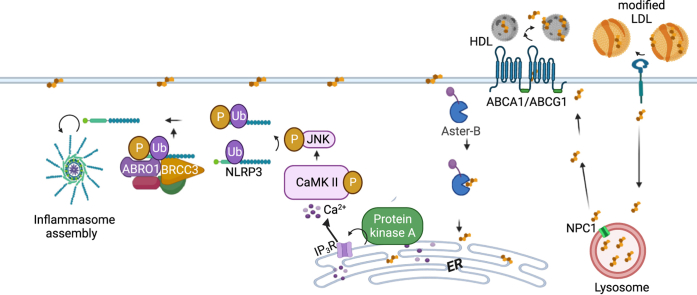

NLRP3 inflammasome activation is often described as a two-step process with priming events increasing the expression of inflammasome components and activation reflecting assembly of the inflammasome (1), binding of the adaptor Apoptosis-associated speck-like protein containing a CARD (ASC) and activation of the effector, Caspase-1 (2). This leads to cleavage of IL-1β, IL-18, and Gasdermin D (GSDMD), which forms membrane pores (3) and permits the secretion of active IL-1β and IL-18. However, the NLRP3 inflammasome is also regulated by post-transcriptional priming and activation events that may involve phosphorylation (4, 5, 6, 7), deubiquitylation, oligomerization, and complex assembly (8). Recent studies have shown that specific phosphorylation of NLRP3 by c-Jun N-terminal Protein Kinase 1 (JNK1) can lead to activation of NLRP3 as a result of deubiquitylation by BRCA1/BRCA2-containing complex 3 (BRCC3), a component of the cytosolic BRISC complex (BRCC3 isopeptidase complex). BRCC3 inhibitors are being developed for suppression of NLRP3 inflammasome activation (9).

The NLRP3 inflammasome has been implicated in the heightened sterile inflammatory responses of several metabolic diseases including obesity and atherosclerosis (10, 11, 12). Atherosclerosis involves a cholesterol-driven macrophage inflammatory response in the arterial wall; however, the mechanisms linking macrophage cholesterol accumulation to inflammation remain poorly understood. Recent clinical studies using IL1β antibodies (13) or colchicine (14, 15) have shown decreased cardiovascular disease (CVD) events, indicating the importance of inflammatory processes and likely inflammasomes in human CVD. In mice, macrophage NLRP3 inflammasome activation worsens atherosclerosis (12), especially when hypercholesterolemia is accompanied by additional pro-inflammatory processes such as diabetes, clonal hematopoiesis (CH), or defective cholesterol efflux (10). NLRP3 inflammasome activation in cholesterol-loaded macrophages has been attributed to the uptake or formation of cholesterol crystals in the endo-lysosomal system, leading to lysosomal membrane damage and release of cathepsins (12, 16). Increased macrophage cholesterol content, because of the deletion of cholesterol efflux-promoting transporters *Abca1* and *Abcg1*, leads to increased NLRP3 inflammasome activation which promotes atherosclerosis (17). However, although there was slightly increased cholesterol crystal formation in *Myl-Abc*^*dko*^ (*LysmCreAbca1*^*fl/fl*^*Abcg1*^*fl/fl*^) macrophages, there was no evidence of lysosomal damage or increased mitochondrial ROS (17, 18). Paradoxically, accumulation of cholesterol in lysosomes due to Niemann-Pick C1 (*Npc1*)-deficiency was found to inhibit NLRP3 inflammasome activation in macrophages suggesting that cholesterol exit from lysosomes and cholesterol trafficking within the cell may be required for inflammasome activation (19). However, macrophages were not examined under cholesterol-loaded conditions that would be relevant to atherosclerosis and a potential pathway of cellular cholesterol trafficking that may be involved in inflammasome activation remains to be elucidated.

In this study, we explored the molecular mechanisms underlying NLRP3 inflammasome activation induced by excessive cellular cholesterol. Our findings indicate that cholesterol trafficking from the plasma membrane (PM) to the endoplasmic reticulum (ER), via Aster-B leads to the activation of calcium/calmodulin-dependent kinase II (CaMKII) and JNK and subsequent NLRP3 deubiquitylation by BRCC3 to promote NLRP3 inflammasome activation. Moreover, a BRCC3 inhibitor or genetic deficiency of *Abro1*, a scaffolding protein with an essential role in the deubiquitinase activity of BRCC3 (20) was shown to reverse increased atherosclerosis and reduce neutrophil extracellular trap formation (NETosis) in atherosclerotic plaques of mice with macrophage cholesterol accumulation due to defective cholesterol efflux. Our findings point to the importance of cholesterol-induced signaling events and post-translational modification of NLRP3 in inflammasome activation.

## Materials and methods

Upon reasonable request to the corresponding authors, the data, analytic methods, and study materials will be made available to other researchers. All supporting data are available within the article and the Expanded Methods in the Data Supplement.

### Mice

All mice except *Abca1*^*fl/fl*^*Abcg1*^*fl/fl*^ and *Abro1*^*−/−*^ were from Jackson Laboratories. *Abca1*^*fl/fl*^*Abcg1*^*fl/fl*^ mice were generated in our laboratory by crossbreeding *Abca1*^*fl/fl*^ mice (kindly provided by Dr John Parks, Wake Forest University) (21) with *Abcg1*^*fl/fl*^ mice (generated at Columbia University), (22) and *Abca1*^*fl/fl*^*Abcg1*^*fl/fl*^ mice were deposited at Jackson Laboratories (stock number 021067). *LysmCreAbca1*^*fl/fl*^*Abcg1*^*fl/fl*^ were generated by crossbreeding *Abca1*^*fl/fl*^*Abcg1*^*fl/fl*^ with *LysmCre* mice (004781), as described before (22). *LysmCreAbca1*^*fl/fl*^*Abcg1*^*fl/fl*^*Abro1*^*−/−*^ mice were generated in our laboratory by crossbreeding *LysmCreAbca1*^*fl/fl*^*Abcg1*^*fl/fl*^ with *Abro1*^*−/−*^ mice (kindly provided by Dr Bin Wang, MD Anderson Cancer Center) (23). *Ldlr*^*−/−*^ mice (002207) and *Tet2*^*−/−*^ mice (023359) were bought from Jackson Laboratories. ASC reporter mice (030744), which constitutively express fluorescent adapter fusion protein (ASC-citrine), was obtained from Jackson Laboratories. *Abca1*^*fl/fl*^*Abcg1*^*fl/fl*^ mice are referred to as control mice, *LysmCreAbca1*^*fl/fl*^*Abcg1*^*fl/fl*^ mice as *Myl-Abc*^*dko*^ mice *and LysmCreAbca1*^*fl/fl*^*Abcg1*^*fl/fl*^*Abro1*^*−/−*^ mice as *Myl-Abc*^*dko*^*Abro1*^*−/−*^ mice. Only female mice were used in atherosclerosis experiments since they are more prone to developing atherogenesis. All mice used for these studies were on a C57BL/6J background and were housed in a specific pathogen-free facility under standard conditions of temperature (about 23 C) with a 12-h light dark cycle and food available ad lib (humidity was not noted). Cages and water were changed every 14–21 days. Fresh food was supplied weekly. For euthanasia, we used CO_2_ inhalation at a rate of 1.9–4.4 L/min for a minimum of 20 min, followed by cervical dislocation. CO_2_ is an asphyxiation agent used for endpoint euthanasia in mice. No anesthetics were used in our studies. All mouse experiments were approved by Institutional Animal Care and Use Committee of Columbia University and were conducted in accordance with the Institutional Animal Care and Use Committee of Columbia University guidelines.

### Bone marrow transplantation

8 weeks old female *Ldlr*^*−/−*^ mice were lethally irradiated with 1 dose of 9.12 Gy from a cesium gamma source. At 24 h after irradiation, the *Ldlr*^*−/−*^ mice were transplanted with 5–10∗10^6^ bone marrow (BM) cells in DMEM containing 2% Fetal bovine serum (FBS) from control*, Myl-ABC*^*dko*^ mice and *Myl-Abc*^*dko*^*Abro1*^*−/−*^ mice. Mice were allowed to recover for 5 weeks after bone marrow (BM) transplantation before Western-type diet (WTD; Harlan Teklad TD88137) feeding for 8 weeks.

### Atherosclerosis studies

*Ldlr*^*−/−*^ mice were transplanted with BM from indicated mice and fed WTD. After 2 weeks of WTD, mice with similar body weights were randomized into two different groups that were respectively injected intraperitoneally with holomycin (1 mg/kg) or vehicle control (DMSO/PBS) every second day for 6 weeks mice then were sacrificed after a total of 8 weeks of WTD. After the indicated period on WTD, mice were sacrificed and hearts were perfused with PBS, isolated, and fixed in phosphate-buffered formalin. Subsequently, hearts were dehydrated and embedded in paraffin, and were cross sectioned throughout the aortic root area. Hematoxylin-eosin staining was performed on the sections and the average from 6 sections for each animal was used to determine lesion size. Lesion size and necrotic area were quantified by morphometric analysis using Image-Pro Plus software (Media Cybernetics). Morphological analysis of fibrous cap thickness was performed with a Masson trichrome stain kit (Maixin-Bio) and was quantified by choosing the largest necrotic core from duplicate sections and measuring the thickness of the thinnest part of the cap.

### Immunofluorescence staining on atherosclerotic plaques

Paraffin-embedded slides were deparaffinized and rehydrated in Trilogy (Cell MARQUE 920P-09). The sections were incubated with primary antibodies for 16 h at 4°C then incubated with secondary antibodies for 30 min. Sections were mounted using DAPI. In all immunofluorescence staining, isotype-matched normal IgG was used as the negative control. For staining of neutrophil extracellular traps, paraffin sections were incubated in Tris-Base EDTA at pH 9.0 (15–20 min; pressure cooker) for antigen retrieval. Then, sections were blocked in PBS containing 10% goat serum for 30 min at 4°C. Subsequently, sections were incubated for 16 h at 4°C with biotinylated myeloperoxidase (MPO) (1:30; R&D systems; BAF3667) or Ly6G (1:200; BioLegend; 127602). When citrullinated histones were stained, sections were concomitantly incubated with Anti-Histone H3 (citrulline R2+R8+R17) antibody (1:300; Abcam; ab5103). For MPO staining, the sections were then incubated with Streptavidin Alexa Fluor 488 (1:200; Invitrogen/Life Technologies; S11223). Anti-rat CF 488A (1:200) was used as secondary antibody for Ly6G staining. When citrullinated histones were stained, sections were concomitantly incubated with Anti-rabbit CF 647 (1:200; Sigma; SAB4600184). Sections were mounted using ProLong Gold Antifade Mountant with DAPI (Thermofisher; P3693) and imaged using a Leica DMI6000B microscope running Leica software. The MPO or Ly6G positive area was quantified using Image-Pro Plus software (Media Cybernetics). The overlap of MPO staining with citrullinated histone staining or Ly6G staining with citrullinated histone staining was assessed as neutrophil extracellular traps and quantified using Image-Pro Plus software (Media Cybernetics).

### Flow cytometry

For quantification of blood myeloid cells, blood was collected by tail bleeding into EDTA-coated tubes and immediately put on ice. Red blood cells (RBCs) were lysed (BD Pharm Lyse, BD Bioscience) and white blood cells were centrifuged, washed, and resuspended in FACS buffer. Cells were stained with a cocktail of antibodies against CD45-APC-Cy7, Ly6-C/G-PerCP-Cy5.5 (BD Pharmingen; 557659 and 561103, respectively), and CD115-APC (eBioscience; 17-1152) in FACS buffer. Monocytes were identified as CD45^hi^CD115^hi^ and further separated into Ly6C^hi^ and Ly6C^lo^ subsets, and neutrophils were identified as CD45^hi^CD115^lo^Ly6C/G^hi^ (Gr-1). For cell surface Toll-like receptor 4 (TLR4) expression analysis, bone marrow–derived macrophages (BMDMs) were cultured in nontissue culture plates (Thermo), and Cellstripper (Corning, 45000-668) was used to gently dislodge them from the plates to avoid damage to cell surface antigens. DMEM with 10% FBS and 1% Penicillin/Streptomycin (P/S) was used to neutralize the Cellstripper, and the BMDMs were centrifuged, washed, and resuspended in Hanks balanced salt solution (HBSS) (0.1% BSA, 5 mmol/L EDTA). The cells were stained for 30 min with a cocktail of antibodies against mouse F4/80-Pacific Blue clone BM8 (Biolegend; 123124, 1:200 dilution) to identify macrophages and TLR4-PE/Cy7 clone SA15-21 (Biolegend; 145408, 1:200 dilution). Flow cytometry was performed using the LSRII (Beckton Dickinson) and data were analyzed using FlowJo software (Beckton Dickinson). Isotype matched normal IgG was used as the control in each flow cytometry assay.

### Isolation of Ly6G^+^ neutrophils and Ly6G^−^CD11b^+^ monocytes

Spleens were mashed on a 40 μm filter and red blood cells were lysed (BD Pharm Lyse, BD Bioscience). First, Ly6G^+^ neutrophils were isolated, using Ly6G^+^ (#130-120-337) coated microbeads, and then, from the same sample, Ly6G^−^CD11b^+^ monocytes/macrophages were isolated, using CD11b^+^ (#130-049-601) coated microbeads (Miltenyi Biotec).

### Blood and serum analysis

Mouse plasma was isolated through centrifugation of blood at 12,000 *g* for 10 min at 4°C. IL-18 plasma levels were measured by ELISA (MBL International, 7625). Total plasma cholesterol was determined using a cholesterol E assay (Wako, 999-02601).

### Bone marrow culture

Bone marrow was flushed from hindlimbs with HBSS and filtered in 60μm cell filters on ice. Cells were centrifuged 800 g for 10 min at 4 C and suspended in DMEM with 10% FBS and 20% L-cell media, 100 U/ml (P/S) (Thermo Fisher, 15140148). Bone marrow cells were then incubated in non-tissue culture treated flasks for 5 days and plated into new tissue culture dishes for 16 h for the indicated assays.

### siRNA-mediated gene silencing

Scrambled siRNA control (Dharmacon, D-001810-10-20), siRNAs against *Gramd1b* (Dharmacon, L-040247-01-0005) and *Brcc3* (Dharmacon, L-060013-01-0010) were transfected into BMDMs using Lipofectamine RNAiMAX (Life Technologies) at 40 nM of siRNA in 24-well or 96 well plates following the manufacturer’s instructions.

### Acetylated LDL preparation

LDL was acetylated (acLDL) by the method of Fraenkel-Conrat as described (24). To summarize, under constant stirring in an ice-water bath, 1 ml of a saturated sodium acetate solution was added to 1 ml of 0.15 M NaCl, which contained 16 mg of LDL protein (24). Next, acetic anhydride was gradually added (total mass 1.5 times that of the protein) while swirling constantly (24), followed by 30 min stirring. (24). Next, the reaction solution was dialyzed against 0.15M NaCl, 0.3 mM EDTA, pH 7.4, for 48 h at 4ºC (24). BMDMs were loaded with vehicle or with modified LDL (acetyl-LDL, acLDL) to promote cholesterol uptake for 16 h.

### Inflammasome assays

BMDMs were preincubated with Lipopolysaccharide (LPS) (20ng/ml), for 3 h then incubated with 2 mM ATP or 10 μg/ml Nigericin for 1 h. At the end of the treatment, cytokines IL-1β or TNF-α in the media were measured using ELISA kits (R&D Systems; DY401 or DY410-05, respectively) and LDH activity in cell media was measured with CyQUANTTM LDH Cytotoxicity Assay (Thermo Fisher Scientific, C20301). Data were normalized to protein concentration of cell lysates. To quantify ASC specks, BMDMs from ASC reporter mice were cultured on Nunc™ Lab-Tek™ Chambered Coverglass (Thermo Fischer, 155411). After indicated treatments, the cells were fixed and visualized imaged using a Leica DMI6000B microscope running Leica software. The average number of specks per field of view was counted. For mechanistical inflammasome assays, treatments of various compounds were done as listed below; 5 μg/ml U18666A compound (Millipore, 662015-10MG) for 48 h prior to LPS, 100 milliunits/ml of Sphingomyelinase (Sigma, S8633-50UN) for 1 h prior to ATP or Nigericin, an Acyl-coenzyme A: cholesterol acyltransferase (ACAT) inhibitor Sandoz 58-035 (Sigma, S9318-5MG) for 30 min prior to LPS, 0.1 mM C2 ceramide (Avanti polar lipids, 860502P-5 mg) for 1 h prior to ATP or Nigericin, 1 μM Xestospongin C (Sigma, 682160-10UG) for 30 min prior to LPS, 1 μM KN-93 (ApexBio, B1306) for 30 min prior to LPS, 1 μM SP600125 (SelleckChem, S1460) for 30 min prior to LPS, 1 μM G5 (Ubiquitin isopeptidase inhibitor I, Sigma, 662125-10MG) for 30 prior to ATP or Nigericin, 25 nM THL (Thiolutin, Tocris, 1567) for 30 prior to ATP or Nigericin, 25 nM HL (Holomycin, Santa Cruz, sc-490291) for 30 prior to ATP or Nigericin, 100 μg/ml Cyclodextrin (CD)-cholesterol (Sigma, C4951) for 1 h prior to ATP or Nigericin. ALOD-4 was purified and used as described before (25, 26). Cholesterol microcrystals are made as described (27) and given to LPS primed BMDMs for additional 6 h.

### Measurement of cholesterol crystals

For imaging of cholesterol crystals, BMDMs were cultured on Nunc™ Lab-Tek™ Chambered Coverglass and were kept at 37°C throughout the experiment and while imaging cholesterol crystals to avoid artificial cholesterol crystal formation. We treated the cells with 5 μg/ml U18666A compound for 48 h and then with 20 ng/ml LPS for 3 h in the presence of LysoTracker (Thermo Fisher, L7528) to visualize the lysosomes. Confocal reflection microscopy was combined with fluorescence microscopy on a Nikon A1RMP multiphoton confocal laser-scanning microscope. Reflection was captured by placement of the detector channel directly over the wavelength of the selected laser channel for reflection light capture and the microscope was set to allow 5–15% of laser light into the collection channel. Fluorescence was simultaneously captured by standard confocal imaging techniques.

### qRT-PCR

To assess mRNA expression, RNA from BMDMs was isolated using a Qiagen RNeasy kit and cDNA was synthesized using kits from Thermo Scientific (Maxima First Strand cDNA synthesis kit; 1642). Initial differences in RNA quantity were corrected by using the housekeeping gene m36B4. SYBR Green Master Mix was from Applied Biosystems (by ThermoScientific; 4385612) and qPCR was run on a StepOne Plus Real-time PCR Systems from Applied Biosystems. qRT-PCR was conducted for specific genes and normalized to m36B4. Primers for qRT-PCR assays in are as follows: *Rplp0* (For: CCTGAAGTGCTCGACATCAC; Rev: CCACAGACAATGCCAGGAC), *Il1b* (For: TGTGAATGCCACCTTTTGACA; Rev: GGTCAA AGGTTTGGAAGCAG), *Srebp2* (For: GCAGCAACGGGACCATTCT; Rev: CCCCATGACTAAGTCCTTCAACT), *Brcc3* (For: GTGCAGGCGGTTCATCTTGA; Rev: AACTCCCCTATACACAGACCC), *Gramd1b* (For: CTGCCGTCCATTGAGATTACG; Rev: TCAGGAACCTGCTCGAATCAT) and *Dusp10* (For: CCA TCT CCT TTA GAC GAC AGG G; Rev: GCT ACC ACT ACC TGG GCT G).

### Immunoprecipitation

To detect NLRP3 ubiquitination, cells were lysed in a lysis buffer (M-PER™ Mammalian Protein Extraction Reagent, Thermo Fisher, 78505). Endogenous NLRP3 was immuno-precipitated with anti-NLRP3 (Adipogen, AG-20B-0014-C100) and Dynabeads™ Protein A Immunoprecipitation Kit (Thermo Fisher, 10006D). To detect NLRP3-BRCC3 interaction, endogenous BRCC3 was immuno-precipitated with anti-BRCC36 (Abcam, ab108411) as described above. Immunoprecipitated complexes were washed in lysis buffer and proteins were eluted in Laemmli buffer (Boston Products, BP-111R).

### Immunoblotting

BMDMs or Ly6G^−^CD11b^+^ monocytes/macrophages were lysed in lysis buffer containing protease/phosphatase inhibitor on ice for 30 min and then centrifuged at 14,000 *g* for 5 min. Protein lysates were separated by 4–20% gradient SDS-PAGE and transferred to nitrocellulose membranes. Then the membranes were blocked with 5% non-fat milk in TBPS-T and incubated with primary antibodies, anti-Caspase-1 (14-9832-82, eBioScience, 1:2,000), anti-GSDMD (Genentech, 1:1,000), anti-NLRP3 (15101S, Cell Signaling, 1:1,000), anti-BRCC3 (Abcam, ab108411, 1:1,000), anti-Ubiquitin (Cell signaling 43124, 1:1,000), anti-p- SAPK/JNK (Thr183/Tyr185) (Cell signaling 4668S, 1:1,000), anti-SAPK/JNK (Cell signaling 9252S, 1:2,000), anti-phospho-CREB (Ser133) (Cell signaling 9198S, 1:1,000), anti-CREB (Cell signaling 9104S, 1:2,000), anti-p-CaMKII (Novus Biologicals, NB300-184, 1:1,000), anti-CaMKII (Novus Biologicals, NBP2-15685, 1:100) and β-actin (Cell signaling 4970S, 1:5,000) at 4°C for 16 h and detected using HRP-conjugated secondary antibodies. The protein levels were quantified using ImageJ. Each phosphorylated or ubiquitylated protein was normalized with its matching total protein for, or pro-form of Caspase-1 and GSDMD. To normalize the treatment group, we used the same factor that established the vehicle or control group as 1, and we averaged the densitometric analyses from all replicates for the control or vehicle group.

### Statistical analysis

All data are presented as means + SEM. The number of mice included in the experiments can be found in the figure legends. Outliers were excluded if the ROUT coefficient Q was <1% as determined by the Prism software. The *t* test was used to define differences between 2 datasets. For statistical analysis of IL-1β secretion, LPS only conditions are excluded from analysis since IL-1β secretion is very low or sometimes not detectable in LPS only conditions as well as we are interested in conditions where inflammasome is activated by ATP, Nigericin or cholesterol crystals. The one-way ANOVA coupled with Tukey’s test for multiple comparisons was used define differences between more than 2 datasets if there is only one variable. The two-way ANOVA (with Sidak’s multiple comparison test) was conducted for where two variables (genotype vs. treatment or diet or siRNA or virus) were being assessed. Two-way ANOVA results are shown next to dot plot with the exact *P*-value of genotype, exact *P*-value of treatment or siRNA used and exact *P*-value of interactions between genotype and treatment or diet or siRNA used. The criterion for significance was set at ∗∗∗∗*P* < 0.0001, ∗∗∗*P* < 0.001, ∗∗ *P* < 0.01, ∗*P* < 0.05. Statistical analyses were performed using GraphPad Prism 8 software.

## Results

### Cholesterol trafficking from PM to ER activates NLRP3 inflammasome

**Fig. 1:**
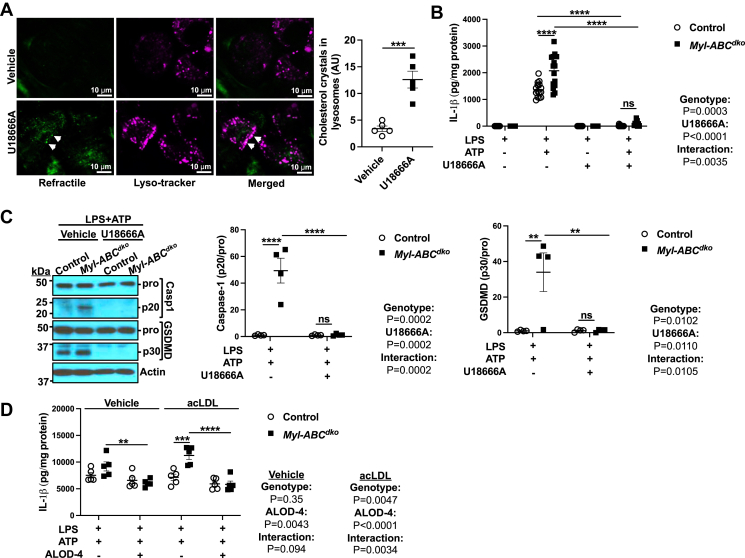
Cholesterol trafficking from plasma membrane to ER increases NLRP3 inflammasome activation. A: Cholesterol crystals in wild-type bone marrow-derived macrophages (BMDMs) treated with vehicle or U18666A (5 μg/ml) for 48 h and primed with LPS (20 ng/ml) for 3 h. Refractile material and lysosomes were assessed using confocal microscopy. B: IL-1β secretion from *Abca1*^*fl/fl*^*Abcg1*^*fl/fl*^ (control) and *LysmCreAbca1*^*fl/fl*^*Abcg1*^*fl/fl*^(*Myl-Abc*^*dko*^) BMDMs that were treated with vehicle or U18666A for 48 h, with LPS for 3 h, and with vehicle or ATP for an additional 1 h to induce inflammasome activation. IL-1β secretion was assessed via ELISA. C: Immunoblot of intracellular Caspase-1 and GSDMD cleavage from (B). D: IL-1β secretion from control and *Myl-Abc*^*dko*^ BMDMs loaded with vehicle or acLDL to promote cholesterol uptake for 16 h. Then, BMDMs were primed with LPS for 3 h and treated with vehicle or ALOD-4 for 1 h and treated with ATP for an additional 1 h. ∗∗∗∗*P* < 0.0001, ∗∗∗*P* < 0.001, ∗∗ *P* < 0.01, ∗*P* < 0.05 by *t* test (A) and two-way ANOVA with Sidak’s multiple comparison test (B–D).

To assess the potential role of lysosomal cholesterol accumulation and cholesterol crystal formation in mediating inflammasome activation, we treated macrophages with acLDL to promote cholesterol uptake and with U18666A to block cholesterol exit from lysosomes (28) and primed with 20 ng/ml lipopolysaccharide (LPS). This led to cholesterol crystal formation in lysosomes (Fig. 1A) and elevated *Srebp2* expression consistent with decreased cholesterol in the ER (supplemental Fig. S1A). We similarly treated control and *LysmCreAbca1*^*fl/fl*^*Abcg1*^*fl/fl*^ (*Myl-Abc*^*dko*^) macrophages (that have increased inflammasome activation and cholesterol crystal formation (17)) and found that U18666A treatment abolished IL-1β secretion (Fig. 1B) as well as cleavage of Caspase-1 and GSDMD into their active forms, in both control and *Myl-Abc*^*dko*^ macrophages (Fig. 1C), while only slightly decreasing *Il1b* mRNA in *Myl-Abc*^*dko*^ macrophages (supplemental Fig. S1B). These findings dissociate cholesterol crystal accumulation from inflammasome activation. Since cholesterol may be transferred from lysosomes to PM prior to movement to the ER (29, 30), acLDL-loaded control and *Myl-Abc*^*dko*^ macrophages were treated with ALOD-4, which sequesters the pool of accessible PM cholesterol and blocks trafficking to ER (25, 26). ALOD-4 treatment suppressed IL-1β secretion and abolished the difference between control and *Myl-Abc*^*dko*^ macrophages (Fig. 1D) while increasing *Il1b* mRNA in *Myl-Abc*^*dko*^ macrophages (supplemental Fig. S1C). These findings suggest a role of cholesterol trafficking from the accessible PM cholesterol pool to the ER in inflammasome activation independent of inflammasome priming.

**Fig. 2:**
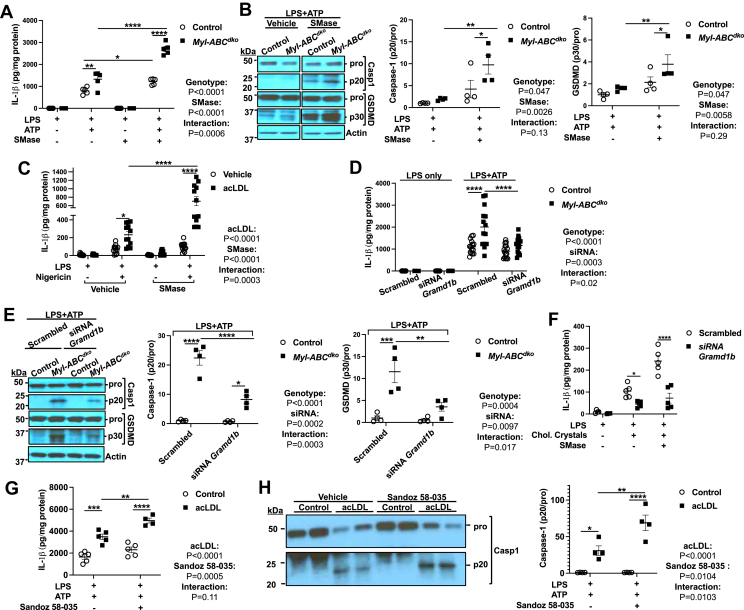
Cholesterol trafficking from plasma membrane to ER via StARD protein complex increases NLRP3 inflammasome activation. A: IL-1β secretion from control and *Myl-Abc*^*dko*^ BMDMs that were treated with LPS for 3 h, vehicle or SMase for 1 h, and with vehicle or ATP for an additional 1 h to induce inflammasome activation. B: Immunoblot of intracellular Caspase-1 and GSDMD cleavage from (A). C: IL-1β secretion from vehicle and acLDL loaded wild-type BMDMs that were primed with LPS for 3 h and treated with vehicle or SMase for 1 h and treated with vehicle or Nigericin for an additional 1 h to induce inflammasome activation. D: IL-1β secretion from control and *Myl-Abc*^*dko*^ BMDMs transfected with control (Scrambled) or siRNA against *Gramd1b* for 48 h. After 48 h of transfection, cells were primed with LPS for 3 h and treated with SMase for 1 h and treated with vehicle or ATP for an additional 1 h. E: Immunoblot of intracellular Caspase-1 and GSDMD cleavage from (D). F: IL-1β secretion from wild-type BMDMs transfected with control (Scrambled) or siRNA against *Gramd1b* for 48 h. After 48 h of transfection, cells were primed with LPS for 3 h and treated with vehicle or cholesterol microcrystals for additional 6 h to induce inflammasome activation. G: IL-1β secretion from vehicle and acLDL loaded wild-type BMDMs that were pre-treated macrophages with vehicle or ACAT inhibitor (Sandoz 58-035) for 30 min and primed with LPS for 3 h and treated with ATP for an additional 1 h to induce inflammasome activation. H: Immunoblot of intracellular Caspase-1 cleavage from (G). ∗∗∗∗*P* < 0.0001, ∗∗∗*P* < 0.001, ∗∗ *P* < 0.01, ∗*P* < 0.05 by *t* test (A) and two-way ANOVA with Sidak’s multiple comparison test.

A distinct pool of PM cholesterol is sequestered by Sphingomyelin (SM) (31). To further assess the role of cholesterol trafficking from PM to ER in inflammasome activation, we treated macrophages with Sphingomyelinase (SMase) to trigger the movement of SM-sequestered cholesterol to the ER (31). When macrophages were primed with LPS and then treated with SMase, *Srebp2* expression was decreased (supplemental Fig. S1D) while *Il1b* mRNA was unaffected (supplemental Fig. S1E). SMase treatment increased IL-1β secretion, Caspase-1, and GSDMD cleavage in both control and *Myl-Abc*^*dko*^ macrophages with a more robust effect in *Myl-Abc*^*dko*^ macrophages (Fig. 2A, B) while having no effect on TLR4 cell surface expression or TNFα secretion (supplemental Fig. S1F, G), suggesting that increased cholesterol in the ER promotes inflammasome activation independent of priming. SMase treatment increased IL-1β secretion in wild-type macrophages loaded with acLDL but not in non-loaded macrophages (Fig. 2C). These findings suggest that subsequent to lysosomal cholesterol loading, increased movement of cholesterol to the ER from both accessible and SM bound pools of plasma membrane cholesterol can promote inflammasome activation.

To further assess the role of PM cholesterol to ER transport and the role of different inflammasomes, we used cholesterol supplementation by cholesterol-methyl-β-cyclodextrin (CD-cholesterol) following LPS priming. CD-cholesterol loading increased IL-1β secretion in response to both ATP and Nigericin (supplemental Fig. S2A). Further, cholesterol supplementation by CD-cholesterol and SMase treatment elevated ASC speck formation, indicating inflammasome assembly, in macrophages from ASC reporter mice (32) (supplemental Fig. S2B). Increased cholesterol biosynthesis and ER cholesterol content have been associated with cholesterol-induced AIM2 inflammasome activation (33) and the AIM2 inflammasome promotes atherosclerosis in *Jak2*^*V617F*^ CH (34). SMase treatment only slightly increased LDH release or IL-1β secretion in response to poly(dA:dT) in *Myl-Abc*^*dko*^ macrophages (supplemental Fig. S2C, D). To clarify whether ceramide formation due to SMase treatment increases inflammasome activation, we supplemented macrophages with ceramide in parallel to SMase treatment. Neither LDH release nor IL-1β secretion was increased by ceramide supplementation in *Abca1/g1* deficient macrophages (supplemental Fig. S2E, F). Similarly, ASC speck formation was induced by SMase or CD-cholesterol supplementation but not ceramide supplementation (supplemental Fig. S2G). These findings further support that SMase promotes NLRP3 inflammasome assembly and activation in cholesterol-loaded macrophages by promoting the movement of cholesterol from PM pools to the ER while having only minor effects on the AIM2 inflammasome.

Because cholesterol is transported from the PM to the ER by the GRAMD1 lipid transfer protein complex (Aster proteins) (35), we next assessed whether knockdown of *Gramd1b* (also known as Aster-B) would affect NLRP3 inflammasome activation in the context of cholesterol loading. Upon *Gramd1b* knockdown (supplemental Fig. S2H), IL-1β secretion, Caspase-1, and GSDMD cleavage were unaffected in control macrophages but were reduced in *Abca1/g1* deficient macrophages to the same level as control macrophages (Fig. 2D, E), while having no effect on TLR4 cell surface expression (supplemental Fig. S2I). Similarly, *Gramd1b* knockdown reduced ASC speck formation induced by acLDL loading to a similar level as controls (supplemental Fig. S2J). To further assess the effects of cholesterol loading we introduced cholesterol in an ethanolic solution, which leads to cholesterol microcrystal formation (24, 27). *Srebp2* mRNA expression was decreased (supplemental Fig. S2K) consistent with elevated cholesterol in the ER. IL-1β secretion was induced by cholesterol crystals, further elevated by SMase treatment, and abolished by *Gramd1b* knockdown (Fig. 2F). Excessive ER cholesterol is converted into cholesteryl esters by ACAT. To further assess the role of ER cholesterol in inflammasome activation, we pre-treated macrophages with an ACAT inhibitor (Sandoz 58-035). ACAT inhibition increased IL-1β secretion and Caspase-1 cleavage in cholesterol-loaded macrophages treated with LPS and ATP (Fig. 2G, H). These results suggest that increased trafficking of cholesterol to the ER via the Aster-B leads to expansion of the ACAT-accessible ER cholesterol pool augmenting activation of the NLRP3 inflammasome.

### ER cholesterol promotes inflammasome activation via a CaMKII-JNK axis

**Fig. 3:**
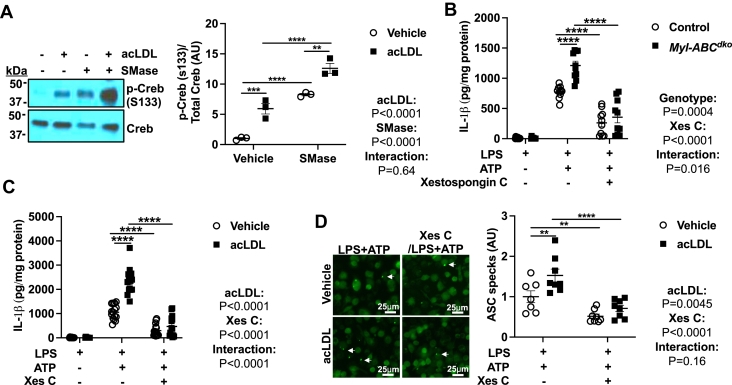
Cholesterol trafficking to ER elevates inflammasome activation via IP3R mediated Ca^2+^ release. A: Immunoblot of Creb (S133) phosphorylation in vehicle and acLDL loaded wild-type BMDMs that were primed with LPS for 3 h and treated with vehicle or SMase for 30 min (B, C) BMDMs were pre-treated with vehicle or Xestospongin C (IP3R blocker) for 30 min and primed with LPS for 3 h and treated with vehicle or ATP for an additional 1 h to induce inflammasome activation. B: IL-1β secretion from control and *Myl-Abc*^*dko*^ BMDMs. C: IL-1β secretion from vehicle and acLDL loaded wild-type BMDMs. D: ASC puncta formation from vehicle and acLDL loaded ASC/citrine BMDMs that were pre-treated with vehicle or Xestospongin C for 30 min and primed with LPS for 3 h and treated with ATP for an additional 1 h to induce inflammasome activation. ∗∗∗∗*P* < 0.0001, ∗∗∗*P* < 0.001, ∗∗ *P* < 0.01, ∗*P* < 0.05 by two-way ANOVA with Sidak’s multiple comparison test.

We next evaluated potential mechanisms linking ER cholesterol accumulation to NLRP3 inflammasome activation. Several human and mouse genetic studies have indicated a role of ER Ca^2+^ mobilization in NLRP3 inflammasome activation (36, 37, 38). Since elevations in ER cholesterol activate Ca^2+^ release from the ER via Protein kinase A (PKA) and inositol triphosphate-3 receptor (IP_3_R) activation (39), we assessed the role of this pathway in NLRP3 inflammasome activation. AcLDL loading and SMase treatment markedly increased phospho-CREB, a marker of PKA activation (Fig. 3A). Moreover, IL-1β secretion in *Abca1/g1* deficient or acLDL loaded macrophages was reduced to the same level as controls upon treatment with Xestospongin C (Xes C), a membrane-permeable blocker of IP_3_-mediated Ca^2+^ release (Fig. 3B, C). Similarly, ASC speck formation in acLDL loaded macrophages was inhibited by Xes C (Fig. 3D). These findings suggest that cholesterol loading of the ER promotes PKA activation and IP3R-mediated Ca^2+^ release, consistent with previous findings (39), leading to increased NLRP3 inflammasome activation.

**Fig. 4:**
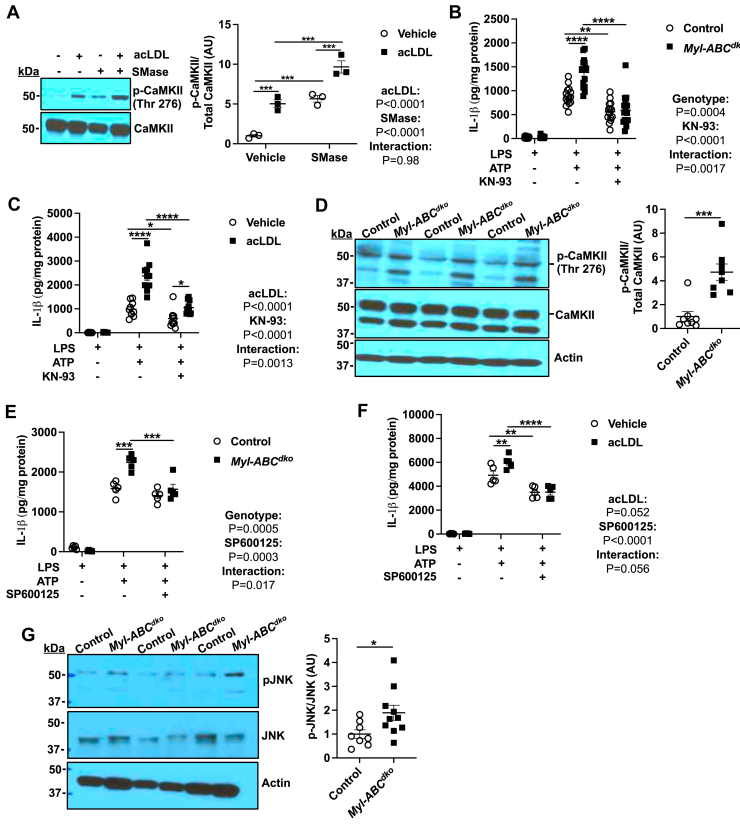
Cholesterol trafficking to ER elevates inflammasome activation via CaMKII/JNK axis. A: Immunoblot of CaMKII (Thr276) phosphorylation in vehicle and acLDL loaded wild-type BMDMs that were primed with LPS for 3 h and treated with vehicle or SMase for 30 min. B, C: BMDMs were pre-treated with vehicle or KN-93 (CaMKII inhibitor) for 30 min and primed with LPS for 3 h and treated with vehicle or ATP for an additional 1 h to induce inflammasome activation. B: IL-1β secretion from control and *Myl-Abc*^*dko*^ BMDMs. C: IL-1β secretion from vehicle and acLDL loaded wild-type BMDMs. D: Immunoblot of CaMKII (Thr276) phosphorylation in Ly6G^−^CD11b^+^ monocytes/macrophages isolated from *Ldlr*^*−/−*^ mice that were transplanted with BM from *Myl-Abc*^*dko*^ mice and fed WTD for 8 weeks. E, F: BMDMs were pre-treated with vehicle or SP600125 (JNK inhibitor) for 30 min and primed with LPS for 3 h and treated with vehicle or ATP for an additional 1 h to induce inflammasome activation. E: IL-1β secretion from control and *Myl-Abc*^*dko*^ BMDMs. F: IL-1β secretion from vehicle and acLDL loaded wild-type BMDMs. G: Immunoblot of JNK (Thr183/Tyr185) phosphorylation in Ly6G^−^CD11b^+^ monocytes/macrophages isolated from *Ldlr*^*−/−*^ mice that were transplanted with BM from *Myl-Abc*^*dko*^ mice and fed WTD for 8 weeks. ∗∗∗∗*P* < 0.0001, ∗∗∗*P* < 0.001, ∗∗ *P* < 0.01, ∗*P* < 0.05 by by *t* test (D, G) and two-way ANOVA with Sidak’s multiple comparison test (A, B, C, E, and F).

Increased ER Ca^2+^ release could potentially lead to activation of CaMKII which in turn could activate JNK-1 (40) leading to activation of the NLRP3 inflammasome via the BRCC3 complex (5, 20). Consistent with this scenario, we showed increased phosphorylation of CaMKII in acLDL-loaded macrophages treated with SMase and in *Abca1/g1* deficient macrophages (Fig. 4A and supplemental Fig. S3A). Treatment with KN-93, a cell-permeable inhibitor of CaMKII, reduced IL-1β secretion in *Abca1/g1* deficient or acLDL-loaded macrophages (Fig. 4B, C) and ASC speck formation in acLDL loaded macrophages (supplemental Fig. S3B). In each case inflammasome activation was not abolished but rather reduced to a similar level to non-cholesterol-loaded controls. Importantly, we also observed increased phosphorylation of CaMKII in Ly6G^−^CD11b^+^ splenic monocytes/macrophages isolated from *Ldlr*^*−/−*^ mice that had been transplanted with bone marrow (BM) from *Myl-Abc*^*dko*^ mice and fed the WTD for 8 weeks (Fig. 4D), confirming in vivo significance. Consistent with activation of JNK downstream of ER Ca^2+^ release and CaMKII activation, we showed increased phosphorylation of JNK in *Abca1/g1* deficient macrophages that was suppressed by Xes C or KN-93 (supplemental Fig. S3C). AcLDL loading and SMase treatment or cholesterol microcrystals also increased pJNK (supplemental Fig. S3D, E). Pre-treatment with SP600125 (a JNK inhibitor) before LPS priming abolished IL-1β secretion in *Abca1/g1* deficient or acLDL loaded macrophages (Fig. 4E, F) and ASC speck formation in acLDL loaded macrophages (supplemental Fig. S3F). Similarly, LPS priming followed by SP600125 treatment during SMase administration, abolished IL-1β secretion induced by SMase in acLDL loaded or *Abca1/g1* deficient macrophages (supplemental Fig. S3G, H), dissociating the effect of the JNK inhibitor from inflammasome priming. Importantly, we also observed increased JNK phosphorylation in Ly6G^−^CD11b^+^ monocytes/macrophages isolated from *Ldlr*^*−/−*^ mice that were transplanted with BM from *Myl-Abc*^*dko*^ mice and fed WTD for 8 weeks (Fig. 4G), supporting that loss of the ABCA1/G1 in macrophages increased JNK activation in vivo. Together these findings support the role of JNK activation downstream of ER Ca^2+^ release in cholesterol induced NLRP3 inflammasome activation.

**Fig. 5:**
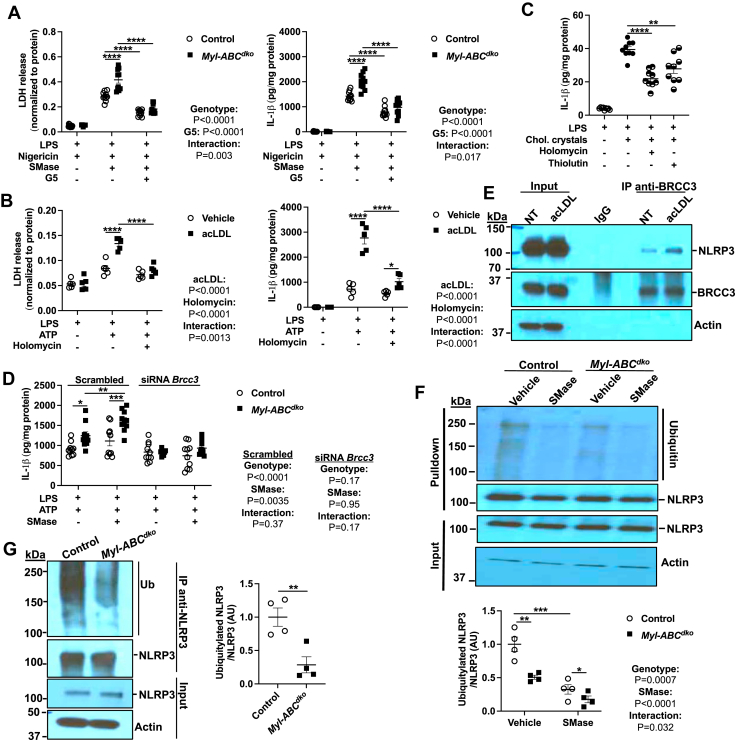
BRCC3-mediated NLRP3 deubiquitylation promotes inflammasome activation by cholesterol accumulation. A: Control and *Myl-Abc*^*dko*^ BMDMs were primed with LPS for 3 h and pre-treated with vehicle or G5 (Deubiquitinase inhibitor) for 30 min and treated with vehicle or Nigericin for an additional 1 h to induce inflammasome activation. LDH release and IL-1β secretion in media were assessed. B: Vehicle and acLDL loaded wild-type BMDMs were primed with LPS for 3 h and treated with vehicle or Holomycin for 30 min and treated with vehicle or ATP for an additional 1 h to induce inflammasome activation. LDH release and IL-1β secretion in media were assessed. C: IL-1β secretion from wild-type BMDMs were primed with LPS for 3 h and treated with vehicle or Holomycin or Thiolutin for 30 min and vehicle or cholesterol microcrystals for additional 6 h to induce inflammasome activation. D: IL-1β secretion from control and *Myl-Abc*^*dko*^ BMDMs transfected with control (Scrambled) or siRNA against *Brcc3* for 48 h. After 48 h of transfection, cells were primed with LPS for 3 h and treated with vehicle or SMase for 1 h and treated with ATP for an additional 1 h. E: Immunoblot of BRCC3 and NLRP3 in cell lysates immunoprecipitated with anti-BRCC3 in vehicle and acLDL loaded wild-type BMDMs primed with LPS for 3 h. F: Immunoblot of NLRP3 ubiquitylation in cell lysates immunoprecipitated with anti-NLRP3 in control and *Myl-Abc*^*dko*^ BMDMs that were treated with LPS for 3 h and vehicle or SMase for 1 h. G: Immunoblot of NLRP3 ubiquitylation in cell lysates immunoprecipitated with anti-NLRP3 in Ly6G^−^CD11b^+^ monocytes/macrophages isolated from *Ldlr*^*−/−*^ mice that were transplanted with BM from *Myl-Abc*^*dko*^ mice and fed WTD for 8 weeks. ∗∗∗∗*P* < 0.0001, ∗∗∗*P* < 0.001, ∗∗ *P* < 0.01, ∗*P* < 0.05 by *t* test (C, G) and two-way ANOVA with Sidak’s multiple comparison test (A, B, D, and G).

In view of the role of JNK in NLRP3 deubiquitylation and activation by BRCC3 (20, 41) we next assessed the role of this mechanism in cholesterol-mediated inflammasome activation. The deubiquitinase (DUB) inhibitor G5 that has been shown to inhibit BRCC3 activation of NLRP3 (41) reduced LDH release and IL-1β secretion in *Abca1/g1* deficient macrophages to the same level as controls (Fig. 5A). Pharmacological targeting of NLRP3 deubiquitylation by thiolutin (THL) and holomycin (HL), a natural methyl derivative of THL was found to suppress NLRP3 activation and some NLRP3-mediated inflammatory processes such as cryopyrin-mediated periodic fever (9). Both THL and HL inhibited LDH release and IL-1β secretion specifically in acLDL-loaded macrophages but not in non-loaded controls (Fig. 5B and supplemental Fig. S4A). Similarly, cholesterol microcrystal induced IL-1β secretion, was suppressed by HL or THL (Fig. 5C). We also used a genetic approach to assess the role of BRCC3. Upon BRCC3 knockdown (supplemental Fig. S4B), IL-1β secretion was unaffected in control macrophages but was reduced to the same level as controls in *Abca1/g1* deficient macrophages (Fig. 5D). Thus, multiple pharmacologic as well as genetic approaches indicate the role of BRCC3 in inflammasome activation by excessive macrophage cholesterol. To verify that NLRP3-BRCC3 complex formation is increased by cholesterol accumulation, we pulled down endogenous BRCC3 in acLDL-loaded BMDMs. AcLDL loading increased the amount of NLRP3 recovered in BRCC3 pulldowns while having no effect on total lysates (Fig. 4E). Moreover, ubiquitinated NLRP3 levels were decreased by SMase treatment in both control and *Abca1/g1* deficient macrophages (Fig. 5F). In line with this finding, ubiquitinated NLRP3 levels were decreased in Ly6G^−^CD11b^+^ monocytes/macrophages isolated from WTD fed *Ldlr*^*−/−*^ mice transplanted with *Myl-Abc*^*dko*^ BM while there was no difference in NLRP3 level in total lysates (Fig. 5G). These findings suggest that increased NLRP3 deubiquitylation mediated through BRCC3/NLRP3 complex formation is a key mechanism underlying NLRP3 inflammasome activation in response to macrophage cholesterol loading.

Recently, we showed that JNK was activated due to the decreased Dual-specificity phosphatase 10 (*Dusp10*) expression in Tet methylcytosine dioxygenase 2 (TET2) deficient macrophages (42). We considered whether the same mechanism could be involved in effects of cholesterol loading. However, treatment of control and *Myl-Abc*^*dko*^ macrophages with U18666A had no effect on the *Dusp10* mRNA expression (supplemental Fig. S5A). Moreover, SMase treatment increased *Dusp10* mRNA expression in control but not *Myl-Abc*^*dko*^ macrophages (supplemental Fig. S5B). We also did not observe any changes in the phosphorylation of CaMKII in a vehicle or acLDL-loaded *Tet2*^*−/−*^ macrophages (supplemental Fig. S5C). These results show that JNK and NLRP3 inflammasome activation induced by increased trafficking of cholesterol to the ER is independent of the changes in the *Dusp10* gene regulation and is dependent on CaMKII activation, which is distinct from NLRP3 inflammasome activation in *Tet2* deficiency.

### BRCC3-mediated NLRP3 deubiquitylation accelerates atherosclerosis

**Fig. 6:**
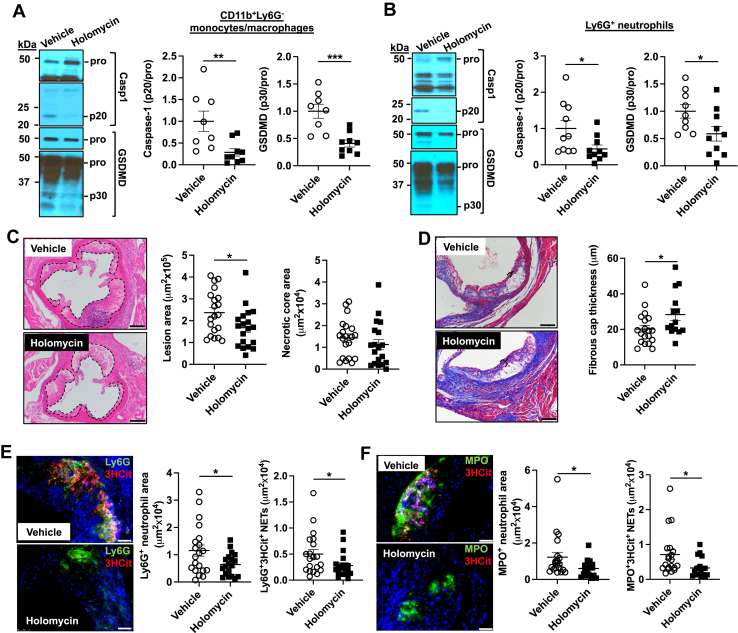
Inhibition of NLRP3 deubiquitylation decreases inflammasome activation and atherosclerosis. *Ldlr*^*−/−*^ mice were transplanted with BM from *Myl-Abc*^*dko*^ mice and fed WTD. After 2 weeks WTD feeding, mice were injected intraperitoneally with HL (1 mg/kg) or vehicle control (DMSO/PBS) for 6 weeks. Mice were sacrificed after a total of 8 weeks of WTD. A, B: Immunoblot of intracellular Caspase-1 and GSDMD cleavage in Ly6G^−^CD11b^+^splenic monocytes/macrophages (A) and splenic Ly6G^+^ neutrophils (B). C: Atherosclerotic lesion area and necrotic core area in the aortic root. Representative images of hematoxylin and eosin (H&E)–stained sections are shown; atherosclerotic plaques are delineated by dashed lines. Scale bars, 200 μm. D: Fibrous cap thickness analysis via a Masson trichrome staining. Bracketed regions show fibrous cap thickness of aortic root plaques. Scale bars, 100 μm. E, F: Neutrophils were stained in atherosclerotic lesions using Ly6G (E) or MPO (F), and Ly6G^+^ (E) or MPO^+^ (F) percentages of lesion size was quantified. Concomitantly, lesions were stained for 3HCit. To assess NETs, the overlap of Ly6G and 3HCit (E) or MPO and 3HCit (F) was quantified as percentage of the total lesion area. Each datapoint represents an individual mouse. Scale bars, 50 μm. N = 20. ∗*P* < 0.05, by *t* test.

To assess the in vivo significance of BRCC3-mediated inflammasome activation, we lethally irradiated *Ldlr*^*−/−*^ mice and transplanted them with *Myl-Abc*^*dko*^ BM cells, followed by treatment with the BRCC3 inhibitor holomycin (HL) or vehicle control. After feeding the WTD for 2 weeks, mice with similar body weights and plasma IL-18 levels were randomized into two different groups that were respectively injected intraperitoneally with HL (1 mg/kg) or vehicle control (DMSO/PBS) every second day for 6 weeks. Mice were sacrificed after 8 weeks of WTD feeding. The administration of HL slightly increased total plasma cholesterol levels (supplemental Fig. S6A) and decreased HDL-cholesterol levels (supplemental Fig. S6B) while having no impact on body weight (supplemental Fig. S6C) in vehicle versus HL group of mice with myeloid *Abca1/g1* deficiency. However, HL administration decreased spleen weight, plasma IL-18 and Ly6C^hi^ monocytes (supplemental Fig. S6D–I) that are known to be increased in *Myl-Abc*^*dko*^ mice and lowered by deletion of inflammasome components (17). HL administration decreased active caspase-1 (p20) and cleaved GSDMD in both Ly6G^−^CD11b^+^ splenocytes (monocytes/macrophages) (Fig. 6A) and Ly6G^+^ splenocytes (neutrophils) (Fig. 6B) and promoted NLRP3 ubiquitylation in Ly6G^−^CD11b^+^ splenocytes consistent with its proposed mechanism of action (9) (supplemental Fig. S6J). These results show that NLRP3 deubiquitylation is key factor in NLRP3 inflammasome activation induced by cholesterol accumulation in vivo. Moreover, HL administration reduced plaque size with no significant change in necrotic core area (Fig. 6C) and improved fibrous cap thickness (Fig. 6D). Inflammasome activation has been associated with neutrophil extracellular trap (NET) formation in atherosclerotic plaques (17), a process that promotes atherothrombosis (43). In atherosclerotic lesions, HL administration significantly decreased both Ly6G^+^ (Fig. 6E) or MPO^+^ (Fig. 6F) neutrophils and NETs measured by Ly6G^+^/3HCit (Fig. 6E) or MPO^+^/3HCit (Fig. 6F) co-staining.

**Fig. 7:**
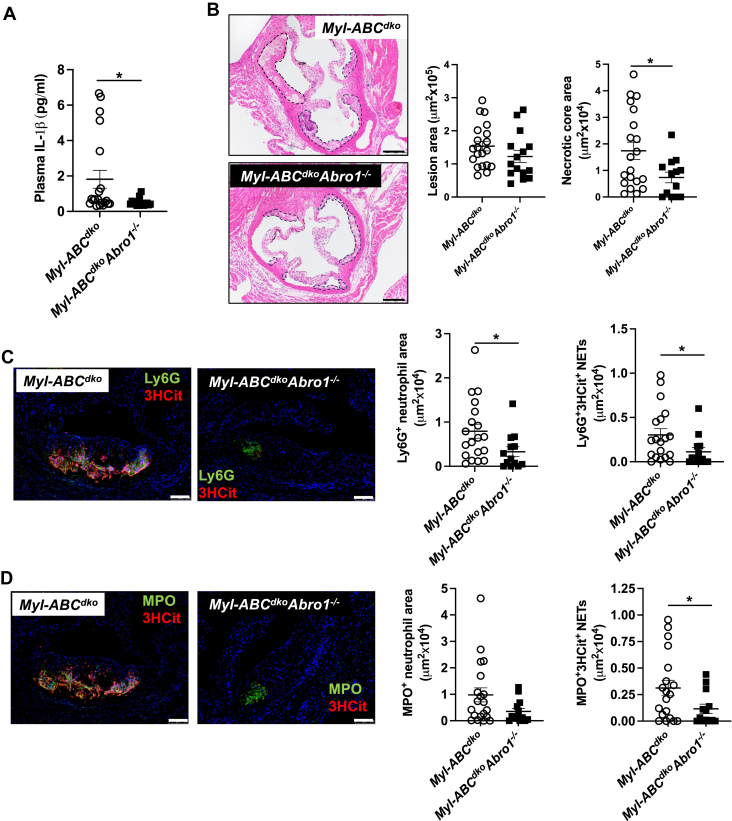
*Abro1* deficiency decreases inflammasome activation and atherosclerosis. *Ldlr*^*−/−*^ mice were transplanted with bone marrow from *Myl-Abc*^*dko*^ or *Myl-Abc*^*dko*^*Abro1*^*−/−*^ mice. After 5 weeks of recovery time, they were fed with WTD and then were sacrificed after a total of 8 weeks of WTD. A: Plasma IL-1β levels. B: Atherosclerotic lesion area and necrotic core area in the aortic root. Representative images of hematoxylin and eosin (H&E)–stained sections are shown; atherosclerotic plaques are delineated by dashed lines. Scale bars, 200 μm. C, D: Neutrophils were stained in atherosclerotic lesions using Ly6G (C) or MPO (D), and Ly6G^+^ (C) or MPO^+^ (D) percentages of lesion size was quantified. Concomitantly, lesions were stained for 3HCit. To assess NETs, the overlap of Ly6G and 3HCit (C) or MPO and 3HCit (D) was quantified as percentage of the total lesion area. Each datapoint represents an individual mouse. Scale bars, 50 μm. N = 20. ∗*P* < 0.05, by *t* test.

To verify the impact of BRCC3-mediated inflammasome activation in vivo, we also employed a genetic approach using the deficiency of the essential BRCC3 co-factor ABRO1 (20). We transplanted *Myl-Abc*^*dko*^ or *Myl-Abc*^*dko*^*Abro1*^*−/−*^ BM into *Ldlr*^−/−^ mice followed by administration of WTD for 8 weeks. *Abro1* deficiency had no effect on body or spleen weight or plasma cholesterol levels (supplemental Fig. S7A–C). Similar to HL administration, Ly6C^hi^ monocytes were significantly decreased by *Abro1* deficiency while other blood leukocytes were unaffected (supplemental Fig. S7D–F). *Abro1* deficiency decreased plasma IL-1β levels (Fig. 7A). Moreover, *Abro1* deficiency tended to reduce plaque size and decreased necrotic core area (Fig. 7B). In atherosclerotic lesions, *Abro1* deficiency significantly decreased Ly6G ^+^ (Fig. 7C) neutrophils and NETs measured by Ly6G^+^/3HCit (Fig. 7C) or MPO^+^/3HCit (Fig. 7D) co-staining.

## Discussion

Our studies point to the importance of cholesterol-induced signaling events and post-translational modification of NLRP3 in NLRP3 inflammasome activation. Cholesterol trafficking from PM to ER, via Aster-B leads to activation of CaMKII/JNK and subsequent NLRP3 deubiquitylation by BRCC3 to promote NLRP3 inflammasome activation. This pathway was activated in response to uptake of modified LDL via the endosomal system, loading with CD-cholesterol or cholesterol microcrystals and by decreased cholesterol efflux in response to knockout of *Abca1* and *Abcg1* and thus appears to be a general mode of response to increased macrophage cholesterol. These findings provide a new mechanism to explain how dyslipidemia involving increased levels of cholesterol-rich lipoproteins and decreased levels of cholesterol efflux promoting HDL (44) can lead to NLRP3 inflammasome activation and increased atherosclerosis.

Following the demonstration of the role of NLRP3 inflammasome activation in atherosclerosis (12), it has become widely accepted that lysosomal damage by cholesterol crystals represents the major mechanism linking excessive macrophage cholesterol to inflammasome activation in atherosclerosis (11, 45). However, we find that cholesterol accumulation in ER rather than lysosomes promotes inflammasome activation and we further define relevant cholesterol trafficking events upstream and signaling events downstream of increased ER cholesterol. While it remains plausible that large cholesterol crystals impact the NRLP3 inflammasome by additional mechanisms in advanced atherosclerotic plaques (12), these mechanisms remain to be clearly defined. Our findings indicate that the progression of early atherosclerosis and NETosis is promoted by a distinct pathway involving ER cholesterol accumulation, CaMKII/JNK activation, and BRCC3-mediated deubiquitylation of NLRP3. Since NETs are formed secondary to NLRP3 activation in myeloid cells (17, 46) this provides a direct connection between cholesterol induced NLRP3 inflammasome activation via BRCC3 in macrophages and plaque NETosis.

Our findings show the importance of increased cholesterol in the ER in promoting NLRP3 inflammasome activation. These findings are consistent with studies by De La Roche *et al.* (19) who showed that decreased cholesterol in the ER due to the NPC mutation led to reduced activation of the NLRP3 inflammasome. Although NLRP3 inflammasome assembly and activation were proposed to be coupled to the SREBP2-SCAP complex near the Golgi (47), our results show that elevations in ER cholesterol increase inflammasome activation, which would be inconsistent with this idea since SREBP2-SCAP formation was shown to be reduced by SMase treatment (35). Nevertheless, the processing of SREBP2 and SREBP2-SCAP formation can occur in response to Nigericin (47), which affects the final step of inflammasome activation. The mechanism we describe here affects earlier post-translational events that prime NLRP3 before the secondary signal comes and the inflammasome assembles. When considering the sequence of the cellular events, decreased SREBP2-SCAP/NLRP3 complex formation could represent a negative feedback mechanism, which may suppress excessive inflammasome activation in the setting of cholesterol overload.

Recently, we showed that HL and *Abro1* deficiency decreased atherosclerosis in mice with TET2 CH in which the NLRP3 inflammasome is an important driver of disease (42, 48). In this study, JNK1 was activated via reduced dephosphorylation reflecting elevated *Dusp10* promoter methylation and decreased *Dusp10* expression in *Tet2*^*−/−*^ deficient macrophages (42). TET2 deficiency acted synergistically with cholesterol loading to increase NLRP3 inflammasome activation (42). However, the mechanisms of the cholesterol effect were not well defined. Here we show that cholesterol loading involves a distinct mechanism in which trafficking of cholesterol from the PM to the ER leads to activation of CAMKII, which increases JNK phosphorylation independent of the changes in the *Dusp10* gene regulation. Our present findings suggest that these distinct mechanisms of JNK activation converge on BRCC3 and NLRP3 in hypercholesterolemia and TET2 CH resulting in synergistic NLRP3 activation (42). Consistent with a role of ER cholesterol/CAMKII, macrophage CaMKII is activated in advanced and symptomatic human carotid atherosclerotic lesions (49), and in mice, myeloid CaMKII deficiency led to suppression of necrotic cores and thicker fibrous caps due to improved efferocytosis in atherosclerotic lesions (49).

The currently available anti-inflammatory therapies that broadly suppress the inflammasome reduced CVD in randomized clinical trials but also resulted in an increase in infectious complications, limiting their clinical adoption (13, 14, 15, 50). Our data suggest a potential therapeutic role of CaMKII and BRCC3 inhibitors that target the cholesterol-induced activation of NLRP3 with limited effects on basal NLRP3 activation. In contrast to complete inhibition of the NLRP3 inflammasome or its products, precise, selective inhibition of dyslipidemia-driven inflammasome activation in atherosclerosis may be less immunosuppressive, and, thus, clinically more acceptable as a therapeutic strategy to reduce CVD risk.

## Data availability

This study includes no data deposited in external repositories. All data are contained within the article.

## Supplemental data

This article contains supplemental data.

## Conflict of interest

The authors declare the following financial interests/personal relationships which may be considered as potential competing interests:

Dr Tall is a member of the SAB of Tensixteen Bio and Beren Therapeutics.

## Supplemental data

Supplemental Figure S1 Cholesterol trafficking from plasma membrane to ER promotes NLRP3 inflammasome activation. A: *Srebp2* mRNA expression in *Abca1*^*fl/fl*^*Abcg1*^*fl/fl*^ (control) and *LysmCreAbca1*^*fl/fl*^*Abcg1*^*fl/fl*^ (*Myl-Abc*^*dko*^) bone marrow derived macrophages (BMDMs) treated with vehicle or U18666A for 48 h and primed with 20 ng/ml LPS for 3 h. B: *Il1b* mRNA expression in control and *Myl-Abc*^*dko*^ BMDMs treated with vehicle or U18666A for 48 h and primed with 20 ng/ml LPS for 3 h. C: *Il1b* mRNA expression in control and *Myl-Abc*^*dko*^ BMDMs primed with 20 ng/ml LPS for 3 h and treated with vehicle or ALOD-4 for 1 h. D, E: *Srebp2* (D) and *Il1b* (E) mRNA expression in control and *Myl-Abc*^*dko*^ BMDMs primed with LPS for 3 h and treated with vehicle or SMase for 1 h. F: Flow cytometry analysis of cell surface TLR4 expression in control and *Myl-Abc*^*dko*^ BMDMs primed with LPS for 3 h and treated with vehicle or SMase for 1 h. G: TNF-α secretion from in control and *Myl-Abc*^*dko*^ BMDMs that were primed with LPS for 3 h and treated with vehicle or SMase for 1 h and treated with vehicle or ATP for an additional 1 h to induce inflammasome activation. ∗∗∗∗*P* < 0.0001, ∗∗∗*P* < 0.001, ∗∗ *P* < 0.01, ∗*P* < 0.05 by two-way ANOVA with Sidak’s multiple comparison test. **Supplemental Figure S2**: Cholesterol trafficking from plasma membrane to ER via StARD protein complex increases NLRP3 inflammasome activation. A: IL-1β secretion from wild-type BMDMs primed with LPS for 3 h and treated with vehicle or cyclodextrin (CD)-cholesterol for 1 h and treated with vehicle or ATP or Nigericin for an additional 1 h to induce inflammasome activation. B: Quantification of ASC puncta formation in BMDMs from ASC/citrine mice that were primed with LPS for 3 h and treated with vehicle or CD-cholesterol for 1 h and treated with vehicle or SMase for 1 h and treated with ATP for an additional 1 h. C: LDH release from control and *Myl-Abc*^*dko*^ BMDMs that were primed with LPS for 3 h and treated with vehicle or SMase for 1 h and treated with vehicle or poly(dA:dT) for additional 6 h to induce AIM2 inflammasome activation. D: IL-1β secretion from (C). E: LDH release from control and *Myl-Abc*^*dko*^ BMDMs that were primed with LPS for 3 h and treated with vehicle or SMase or Ceramide for 1 h and treated with vehicle or ATP for an additional 1 h. F: IL-1β secretion from (E). G: ASC puncta formation in BMDMs from ASC/citrine mice that were primed with LPS for 3 h and treated with vehicle or SMase or CD-cholesterol or Ceramide for 1 h and treated with ATP for an additional 1 h. H: *Gramd1b* mRNA expression in BMDMs transfected with control (Scrambled) or siRNA against *Gramd1b* for 48 h. I: Flow cytometry analysis of cell surface TLR4 expression in control and *Myl-Abc*^*dko*^ BMDMs transfected with control (Scrambled) or siRNA against *Gramd1b* for 48 h and primed with LPS for 3 h. J: Quantification of ASC puncta formation in vehicle and acLDL loaded ASC/citrine BMDMs upon *Gramd1b* knockdown. After 48 h of transfection, macrophages were primed with LPS for 3 h and treated with ATP for additional 1 h. K: *Srebp2* mRNA expression in BMDMs primed with LPS for 3 h and treated with vehicle or cholesterol crystals for 3 h. Arrows show ASC specks. Scale bars, 25 μm. ∗∗∗∗*P* < 0.0001, ∗∗∗*P* ≤ 0.001, ∗∗*P* ≤ 0.01, ∗*P* ≤ 0.05, by t-test (A, G, H and K) and by two-way ANOVA with Sidak’s multiple comparison test (B, C, D, E, F, I, and J). **Supplemental Figure S3**: Cholesterol trafficking to ER elevates inflammasome activation via IP3R/CaMKII/JNK axis. A: Immunoblot of CaMKII (Thr276) phosphorylation in control and *Myl-Abc*^*dko*^ BMDMs that were primed with LPS for 3 h. B: ASC puncta formation from vehicle or acLDL loaded ASC/citrine BMDMs that were pre-treated with vehicle or KN-93 (CaMKII inhibitor) for 30 min and primed with LPS for 3 h and treated with ATP for an additional 1 h to induce inflammasome activation. C: Immunoblot of JNK (Thr183/Tyr185) phosphorylation in control and *Myl-Abc*^*dko*^ BMDMs that were pre-treated with vehicle or Xestospongin C (IP3R blocker) or KN-93 for 30 min and primed with LPS. D: Immunoblot analysis of JNK (Thr183/Tyr185) phosphorylation in vehicle or acLDL loaded wild-type BMDMs that were primed with LPS for 3 h and treated with vehicle or SMase for 30 min. E: Immunoblot of JNK (Thr183/Tyr185) and CaMKII (Thr276) phosphorylation in control BMDMs that were primed with LPS for 3 h and treated with vehicle or cholesterol crystals for 6 h. F: ASC puncta formation from vehicle or acLDL loaded ASC/citrine BMDMs that were pre-treated with vehicle or SP600125 (JNK inhibitor) for 30 min and primed with LPS for 3 h and treated with ATP for an additional 1 h to induce inflammasome activation. G, H: BMDMs were primed with LPS for 3 h and treated with vehicle or SMase 1 h and vehicle or SP600125 and treated with vehicle or ATP for an additional 1 h. G: IL-1β secretion from control and *Myl-Abc*^*dko*^ BMDMs. H: IL-1β secretion from vehicle and acLDL loaded wildtype BMDMs. Arrows show ASC specks. Scale bars, 25 μm. ∗∗∗∗*P* < 0.0001, ∗∗∗*P* < 0.001, ∗∗ *P* < 0.01, ∗*P* < 0.05 by by *t*-test (E), two-way ANOVA with Sidak’s multiple comparison test (B, D, and F) and two-way ANOVA coupled with Dunnett’s test for multiple comparisons against LPS+ATP (G and H). **Supplemental Figure S4**: BRCC3-mediated NLRP3 deubiquitylation inflammasome activation via NLRP3 deubiquitylation by cholesterol accumulation. A: Vehicle and acLDL loaded wild-type BMDMs were primed with LPS for 3 h and pre-treated with vehicle or Thiolutin for 30 min and treated with vehicle or ATP for an additional 1 h to induce inflammasome activation. LDH release and IL-1β secretion were assessed in cell media. B: *Brcc3* mRNA expression in BMDMs transfected with control (Scrambled) or siRNA against *Brcc3* for 48 h. ∗∗∗∗*P* < 0.0001, ∗∗∗*P* < 0.001, ∗∗ *P* < 0.01, ∗*P* < 0.05 by two-way ANOVA with Sidak’s multiple comparison test (A) and *t*-test (B). **Supplemental Figure S5**: Cholesterol trafficking to ER elevates inflammasome activation independent of DUSP10. A: *Dusp10* mRNA expression in control and *Myl-Abc*^*dko*^ BMDMs treated with vehicle or U18666A for 48 h and primed with 20 ng/ml LPS for 3 h. B: *Dusp10* mRNA expression in control and *Myl-Abc*^*dko*^ BMDMs primed with LPS for 3 h and treated with vehicle or SMase for 1 h. C: Immunoblot of CaMKII (Thr276) phosphorylation in vehicle or acLDL loaded control and *Tet2*^*−/−*^ BMDMs that were primed with LPS for 3 h and treated with vehicle or SMase for 30 min. ∗∗∗∗*P* < 0.0001, ∗∗∗*P* < 0.001, ∗∗ *P* < 0.01, ∗*P* < 0.05 by two-way ANOVA with Sidak’s multiple comparison test. **Supplemental Figure S6**: Inhibition of NLRP3 deubiquitylation decreases NETosis and atherosclerosis. *Ldlr*^*−/−*^ mice were transplanted with bone marrow from *Myl-Abc*^*dko*^ mice and fed WTD for 2 weeks. After feeding the WTD for 2 weeks, mice were injected intraperitoneally with Holomycin (HL) (1 mg/kg) or vehicle control (DMSO/PBS) for 6 weeks mice then were sacrificed after a total of 8 weeks of WTD. A: Plasma total cholesterol analysis. B: Plasma HDL cholesterol analysis. C: Body weight (D) Spleen weight. E: Increases in plasma IL-18 after HL administration. F: ELISA analysis of plasma IL-18 after HL administration. G: Flow cytometry analysis of blood neutrophils (% of CD45^+^ cells). H: Flow cytometry analysis of blood Ly6C^low^ monocytes (% of CD45^+^ cells). I: Flow cytometry analysis of blood Ly6C^hi^ monocytes (% of CD45^+^ cells). J: Immunoblot of NLRP3 deubiquitylation in cell lysates immunoprecipitated with anti-NLRP3 in Ly6G^−^CD11b^+^ monocytes/macrophages. Each datapoint represents an individual mouse. ∗∗∗∗*P* < 0.0001, ∗∗∗*P* < 0.001, ∗∗ *P* < 0.01, ∗*P* < 0.05 by t-test. **Supplemental Figure S7**: *Abro1* deficiency decreases inflammasome activation and atherosclerosis. *Ldlr*^*−/−*^ mice were transplanted with bone marrow from *Myl-Abc*^*dko*^ or *Myl-Abc*^*dko*^*Abro1*^*−/−*^ mice. After 5 weeks of recovery time, they were fed with WTD and then were sacrificed after a total of 8 weeks of WTD. A: Body weight. B: Plasma HDL cholesterol analysis. C: Spleen weight. D: Flow cytometry analysis of blood neutrophils (% of CD45^+^ cells). E: Flow cytometry analysis of blood Ly6C^low^ monocytes (% of CD45^+^ cells). F: Flow cytometry analysis of blood Ly6C^hi^ monocytes (% of CD45^+^ cells). Each datapoint represents an individual mouse. ∗∗∗∗*P* < 0.0001, ∗∗∗*P* < 0.001, ∗∗ *P* < 0.01, ∗*P* < 0.05 by *t*-test.
