# Supplementary material for: Cholesterol trafficking to the ER leads to the activation of CaMKII/JNK/NLRP3 and promotes atherosclerosis
Source: J Lipid Res. 2024 Mar 22;65(4):100534. doi: 10.1016/j.jlr.2024.100534 (PMC11031842; doi:10.1016/j.jlr.2024.100534)

Supplementary Figure 1

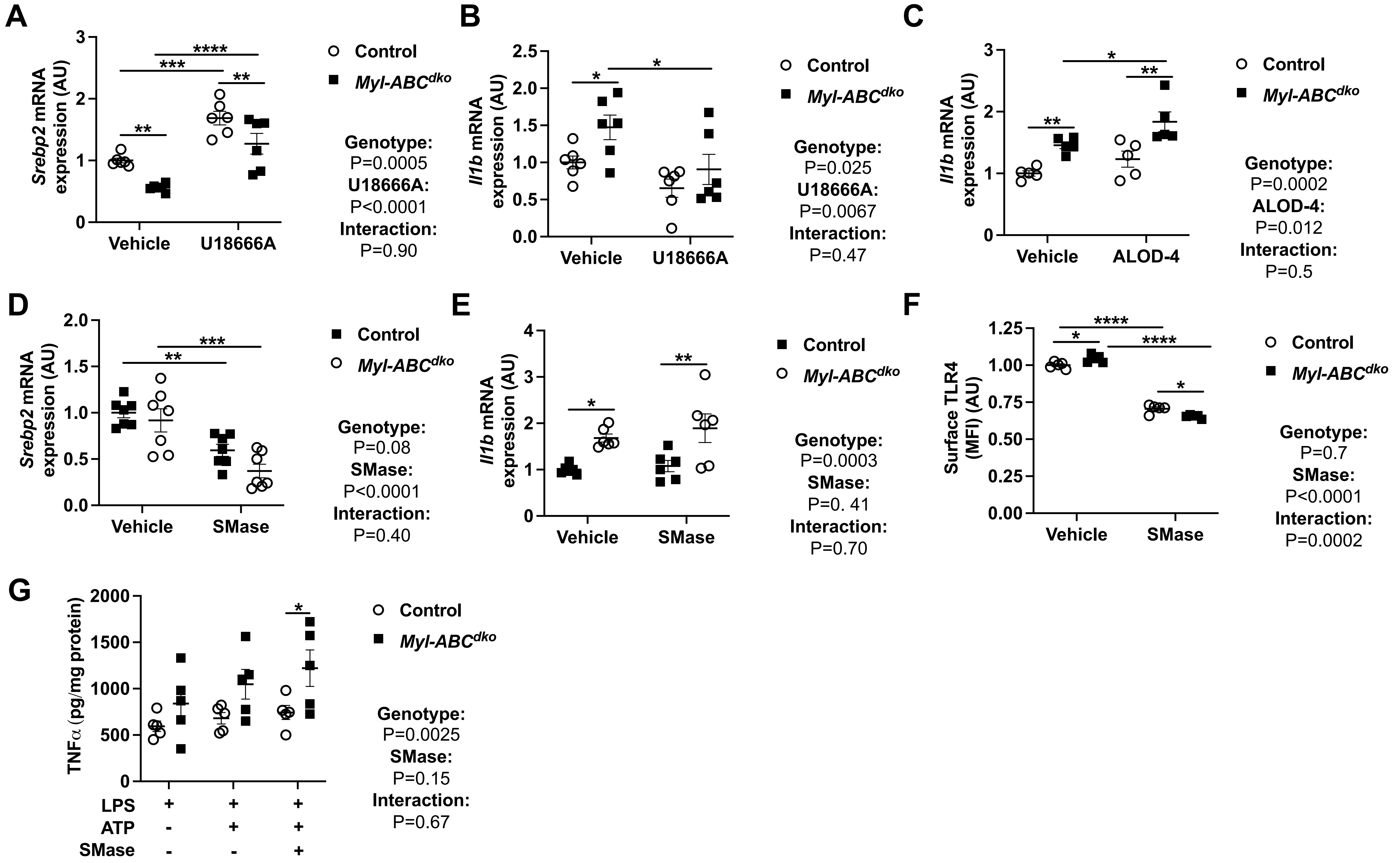

Supplementary Figure 2

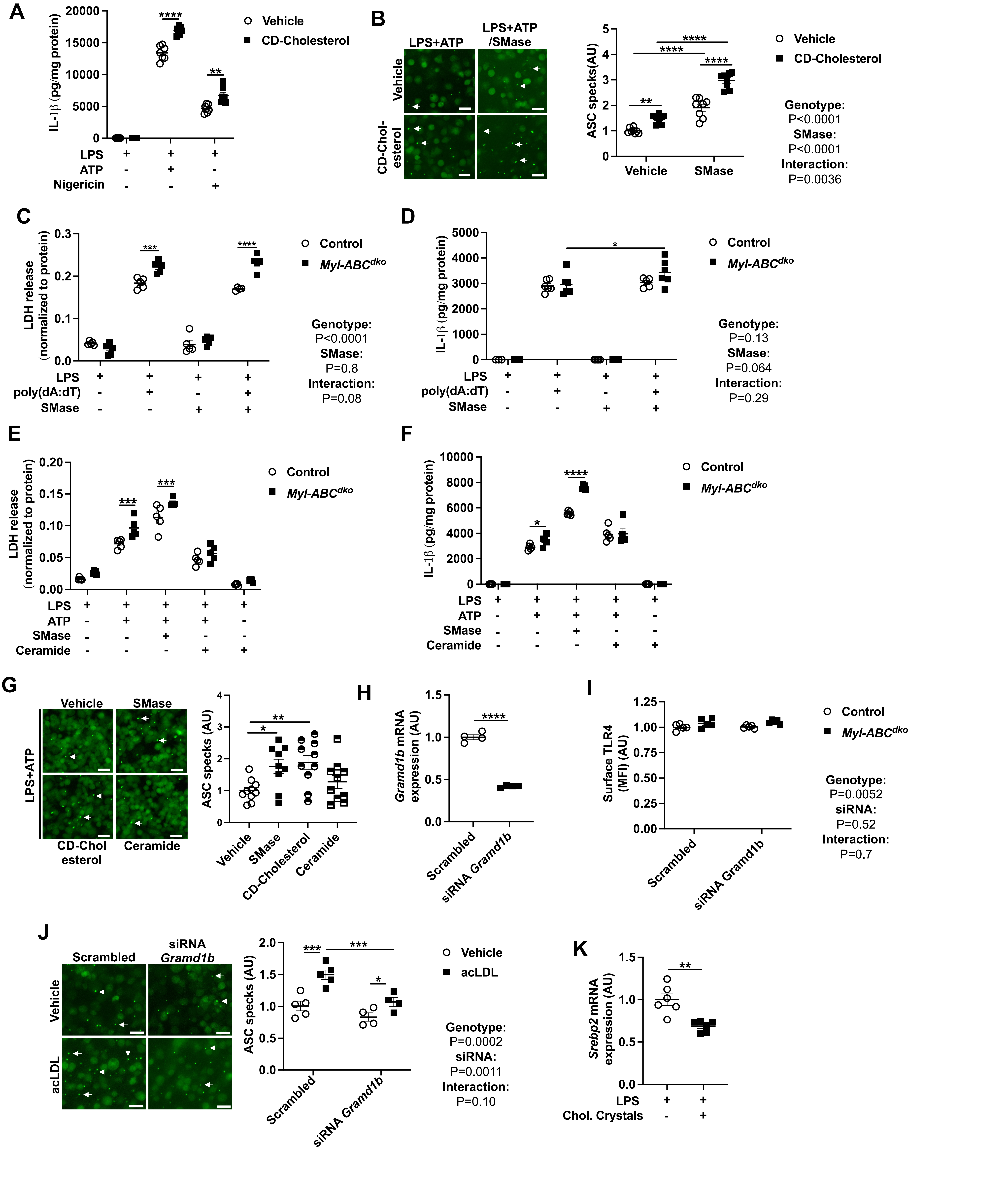

Supplementary Figure 3

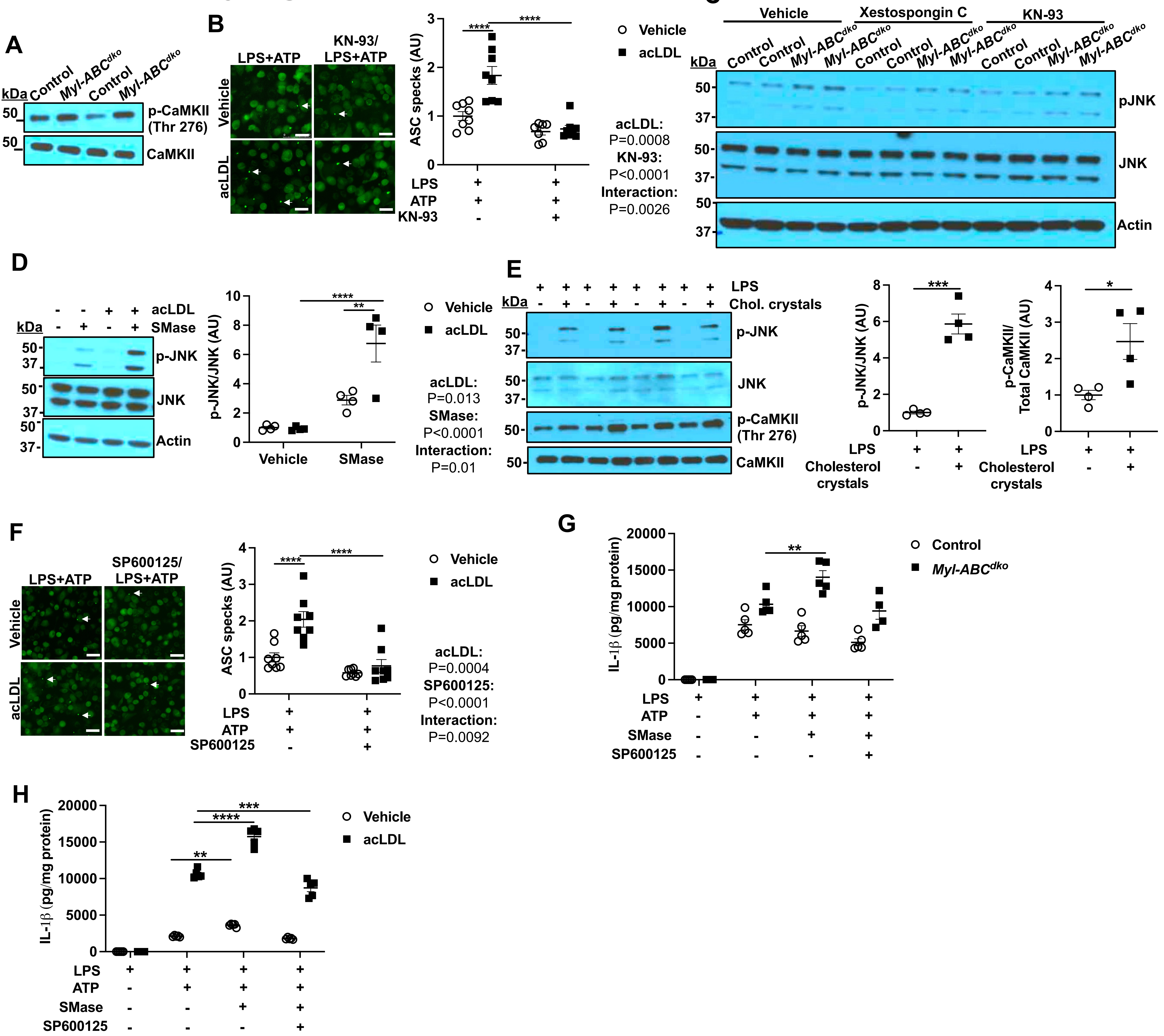

Supplementary Figure 4

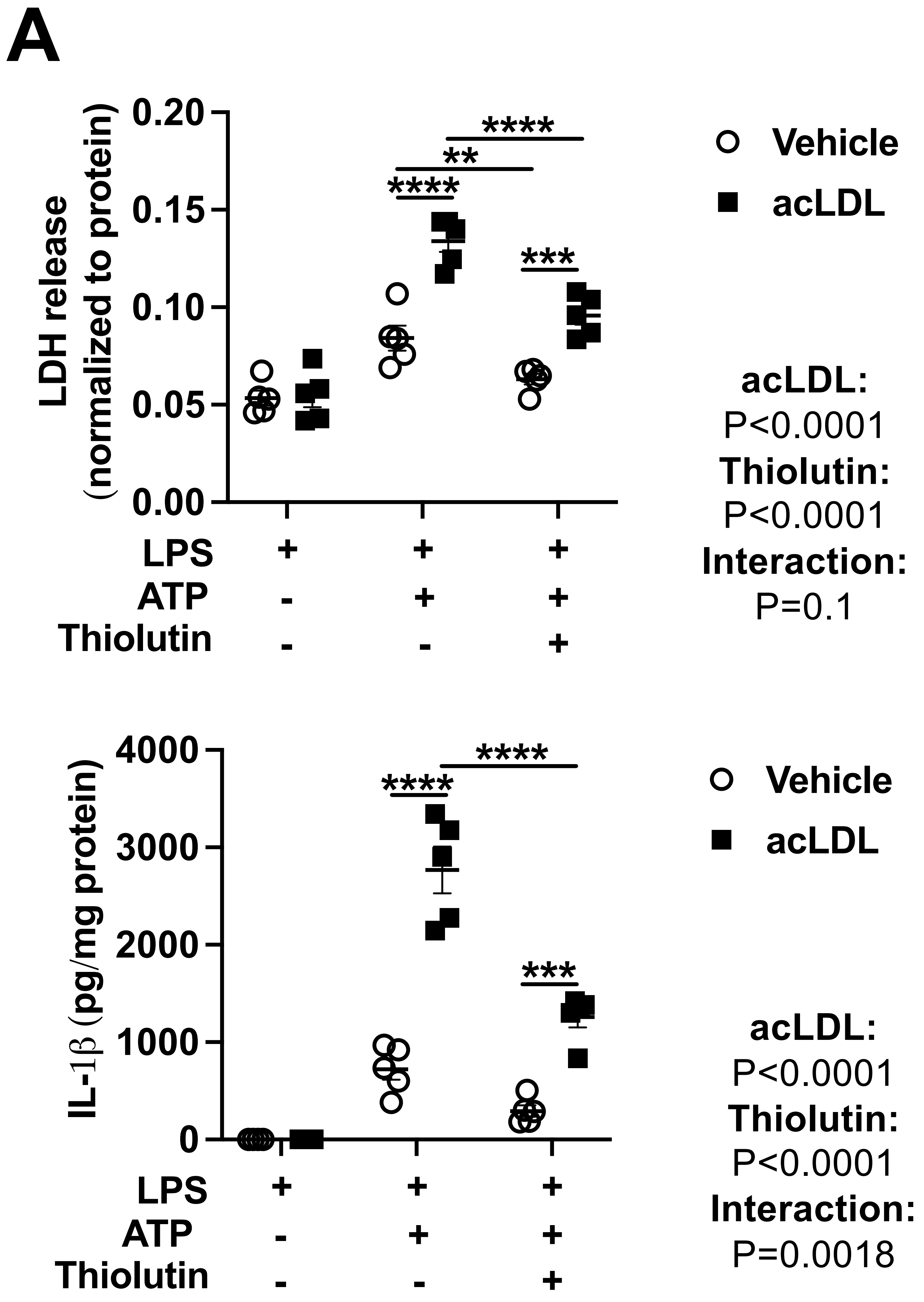

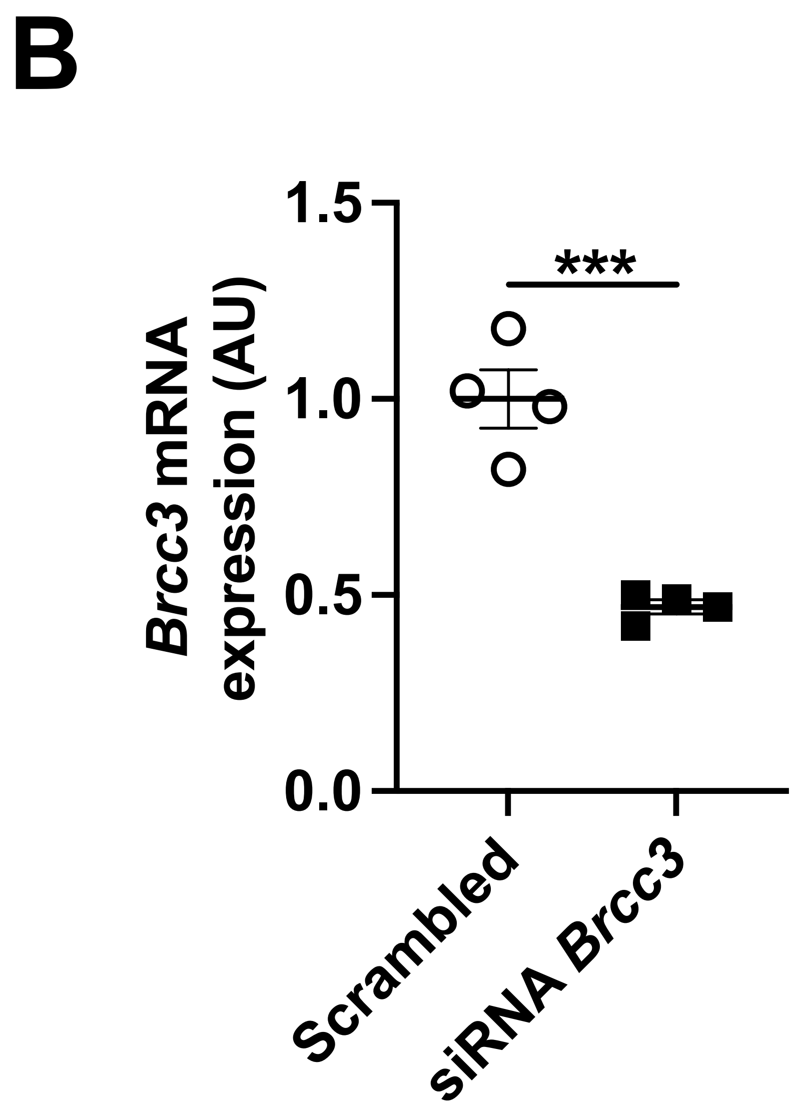

Supplementary Figure 5

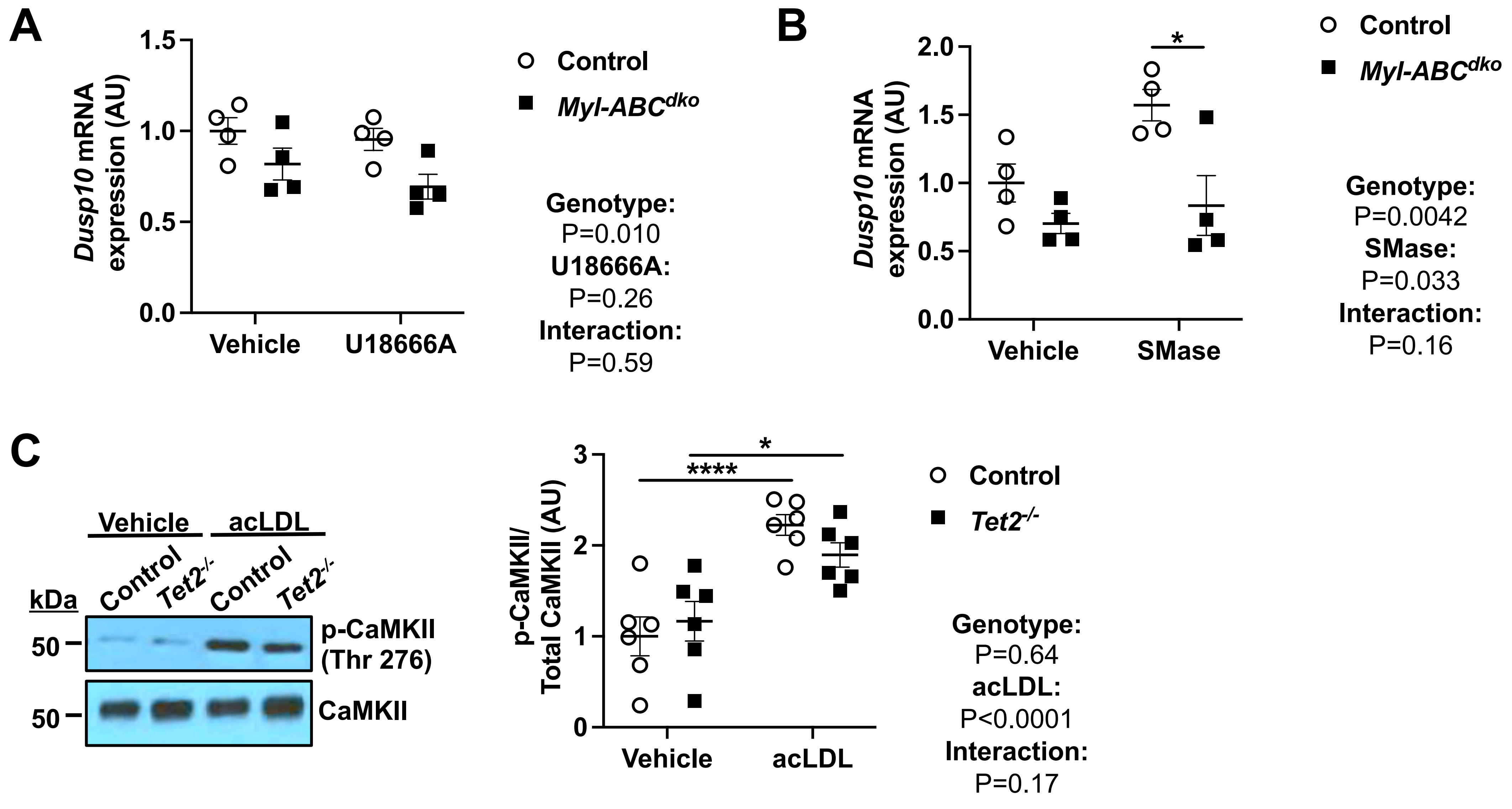

Supplementary Figure 6

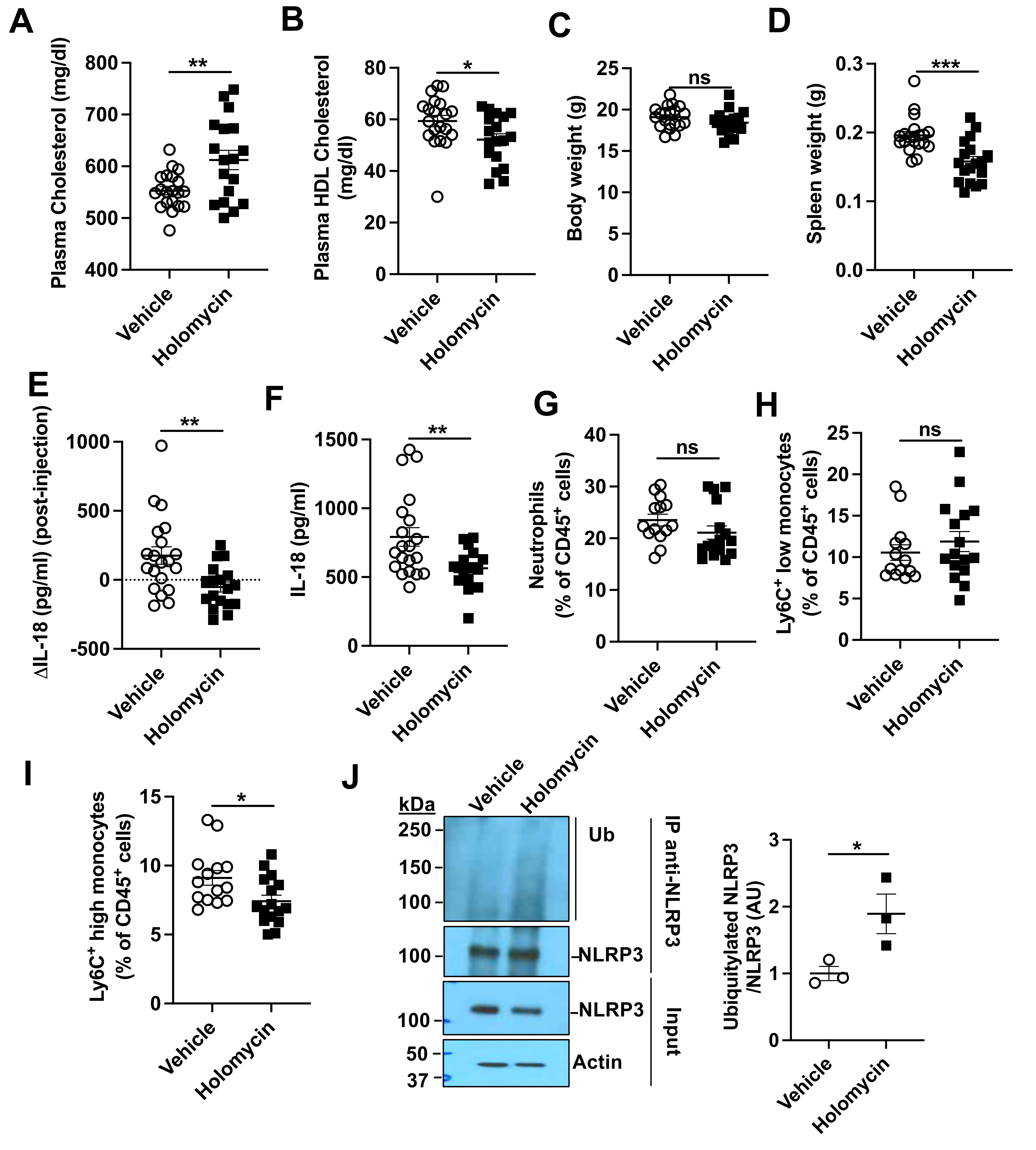

Supplementary Figure 7

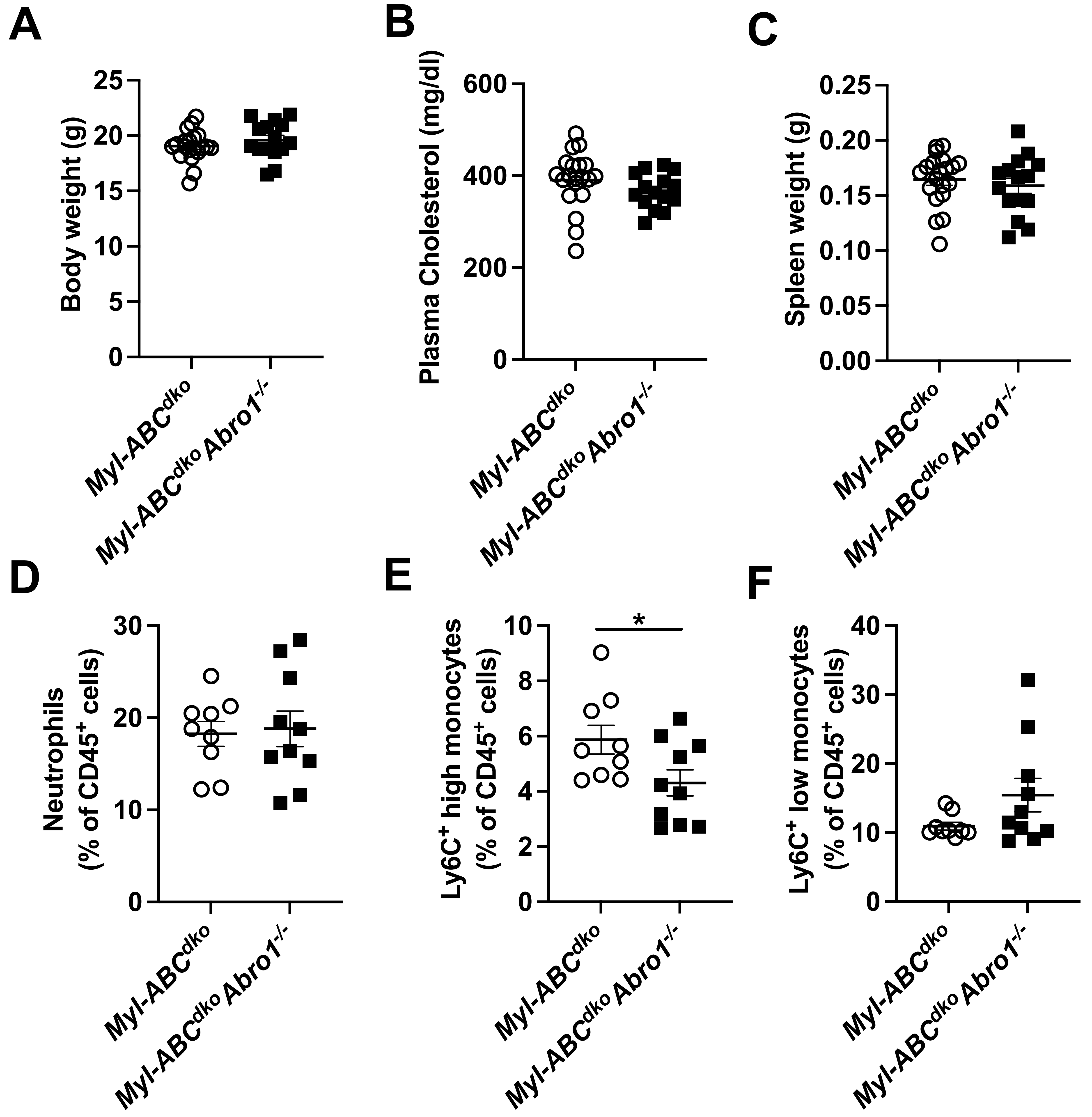

Supplement: Supplemental Figure S1 — Cholesterol trafficking from plasma membrane to ER promotes NLRP3 inflammasome activation. A: Srebp2 mRNA expression in Abca1fl/flAbcg1fl/fl (control) and LysmCreAbca1fl/flAbcg1fl/fl (Myl-Abcdko) bone marrow derived macrophages (BMDMs) treated with vehicle or U18666A for 48 h and primed with 20 ng/ml LPS for 3 h. B: Il1b mRNA expression in control and Myl-Abcdko BMDMs treated with vehicle or U18666A for 48 h and primed with 20 ng/ml LPS for 3 h. C: Il1b mRNA expression in control and Myl-Abcdko BMDMs primed with 20 ng/ml LPS for 3 h and treated with vehicle or ALOD-4 for 1 h. D, E: Srebp2 (D) and Il1b (E) mRNA expression in control and Myl-Abcdko BMDMs primed with LPS for 3 h and treated with vehicle or SMase for 1 h. F: Flow cytometry analysis of cell surface TLR4 expression in control and Myl-Abcdko BMDMs primed with LPS for 3 h and treated with vehicle or SMase for 1 h. G: TNF-α secretion from in control and Myl-Abcdko BMDMs that were primed with LPS for 3 h and treated with vehicle or SMase for 1 h and treated with vehicle or ATP for an additional 1 h to induce inflammasome activation. ∗∗∗∗P < 0.0001, ∗∗∗P < 0.001, ∗∗ P < 0.01, ∗P < 0.05 by two-way ANOVA with Sidak’s multiple comparison test. Supplemental Figure S2: Cholesterol trafficking from plasma membrane to ER via StARD protein complex increases NLRP3 inflammasome activation. A: IL-1β secretion from wild-type BMDMs primed with LPS for 3 h and treated with vehicle or cyclodextrin (CD)-cholesterol for 1 h and treated with vehicle or ATP or Nigericin for an additional 1 h to induce inflammasome activation. B: Quantification of ASC puncta formation in BMDMs from ASC/citrine mice that were primed with LPS for 3 h and treated with vehicle or CD-cholesterol for 1 h and treated with vehicle or SMase for 1 h and treated with ATP for an additional 1 h. C: LDH release from control and Myl-Abcdko BMDMs that were primed with LPS for 3 h and treated with vehicle or SMase for 1 h and treated with vehicle or poly(dA:dT) f [file mmc1.pdf]
